# Fin whales of the Great Bear Rainforest: *Balaenoptera physalus velifera* in a Canadian Pacific fjord system

**DOI:** 10.1371/journal.pone.0256815

**Published:** 2021-09-03

**Authors:** Eric M. Keen, James Pilkington, Éadin O’Mahony, Kim-Ly Thompson, Benjamin Hendricks, Nicole Robinson, Archie Dundas, Linda Nichol, Hussein M. Alidina, Hermann Meuter, Chris R. Picard, Janie Wray

**Affiliations:** 1 North Coast Cetacean Society, Alert Bay, British Columbia, Canada; 2 Environmental Studies, Sewanee: The University of the South, Sewanee, TN, United States of America; 3 Environmental Studies, University of Victoria, Victoria, British Columbia, Canada; 4 Pacific Biological Station, Fisheries and Oceans Canada, Nanaimo, British Columbia, Canada; 5 Scottish Oceans Institute, University of St Andrews, St Andrews, United Kingdom; 6 SoundSpace Analytics, Cumberland, British Columbia, Canada; 7 Gitga’at Oceans and Lands Department, Hartley Bay, British Columbia, Canada; 8 WWF-Canada, Victoria, British Columbia, Canada; 9 Pacific Whale Society, Hartley Bay, British Columbia, Canada; 10 Pacific Orca Society, Alert Bay, British Columbia, Canada; Wildlife Conservation Society Canada, CANADA

## Abstract

Fin whales (*Balaenoptera physalus*) are widely considered an offshore and oceanic species, but certain populations also use coastal areas and semi-enclosed seas. Based upon fifteen years of study, we report that Canadian Pacific fin whales (*B*. *p*. *velifera*) have returned to the Kitimat Fjord System (KFS) in the Great Bear Rainforest, and have established a seasonally resident population in its intracoastal waters. This is the only fjord system along this coast or elsewhere in which fin whales are known to occur regularly with strong site fidelity. The KFS was also the only Canadian Pacific fjord system in which fin whales were commonly found and killed during commercial whaling, pointing to its long-term importance. Traditional knowledge, whaling records, and citizen science databases suggest that fin whales were extirpated from this area prior to their return in 2005–2006. Visual surveys and mark-recapture analysis documented their repopulation of the area, with 100–120 whales using the fjord system in recent years, as well as the establishment of a seasonally resident population with annual return rates higher than 70%. Line transect surveys identified the central and outer channels of the KFS as the primary fin whale habitat, with the greatest densities occurring in Squally Channel and Caamaño Sound. Fin whales were observed in the KFS in most months of the year. Vessel- and shore-based surveys (27,311 km and 6,572 hours of effort, respectively) indicated regular fin whale presence (2,542 detections), including mother-calf pairs, from June to October and peak abundance in late August–early September. Seasonal patterns were variable year-to-year, and several lines of evidence indicated that fin whales arrived and departed from the KFS repeatedly throughout the summer and fall. Additionally, we report on the population’s social network and morphometrics. These findings offer insights into the dynamics of population recovery in an area where several marine shipping projects are proposed. The fin whales of the Great Bear Rainforest represent a rare exception to general patterns in this species’ natural history, and we highlight the importance of their conservation.

## Introduction

The fin whale, *Balaenoptera physalus* (Linnaeus, 1758) occurs throughout the world’s oceans within temperate and subpolar ecosystems [1]. Decades of commercial harvest decimated the global population of this species by as much as 70%, but most populations appear to be recovering [2]. Today, fin whale populations vary from ‘Endangered’ to ‘Vulnerable’, and these statuses are currently under review at various national and international levels [e.g., 2–4]. The north Pacific subspecies, *B*. *p*. *velifera* [5], is managed multi-nationally as several stocks, including three along the west coast of North America [3, 4]. Among these stocks, migratory patterns and rates of interchange remain poorly understood, as do the subspecies’ population structure [5–7]. Fin whales are widely considered an offshore and oceanic species [1, 8–10]. In the northeast Pacific, systematic surveys have found the greatest numbers of fin whales in waters more than 50 nautical miles (nmi) offshore [3, 4, 11]. Similar distributions have been observed from systematic surveys in the North Atlantic and Southern Oceans [1]. These modern-day distributions reflect the offshore concentrations of fin whale catches during 20^th^ century commercial whaling [6, 12]. In several ocean basins, large-scale offshore movements are hypothesized to play a part in this species’ migratory behaviors [1, 13].

However, noteworthy inshore exceptions occur. Resident populations of fin whales have been identified within semi-enclosed seas, such as the Mediterranean Sea [10, 14] and the Gulf of California [15, 16], where site fidelity occurs and gene flow between other habitats is limited. Site fidelity has also been observed along discrete portions of the continental shelf, such as Southern California Bight [7]. Use of coastal areas has also been documented in the Gulf of Alaska [17], the Gulf of St. Lawrence [18–20], the Salish Sea [21, 22], the Bay of Fundy [23], and elsewhere in Canada’s maritime provinces [24]. These exceptions to an otherwise offshore distribution are noteworthy in the natural history of the fin whale as a species, and may prove relevant to the conservation and management of certain stocks. Compared with the open ocean, the coastal, enclosed, and inland habitats utilized by certain fin whale populations are dynamic, productive, and strongly influenced by terrigenous nutrients and human activities [25]. Coastal ecosystems differ in their trophic structure, food web composition, and seasonality of production [26]. Physical processes unique to shallow seas, such as tidal forcing, internal waves, and the interactions between bottom currents and seafloor features, tend to aggregate planktonic organisms in ways that can be exploited by large suspension feeders like the fin whale [23]. The utilization of both coastal and offshore areas allows fin whales to access a wide range of resources and may confer long-term resilience to environmental perturbations, as has been found in other ecologically plastic predators that use disparate systems [27, 28]. In light of this, it is unfortunate that coastal habitats are now the most degraded and disturbed of marine systems [29]. Most of the world’s fishing effort occurs above the continental shelf [30], and anthropogenic debris and chemical pollutants are concentrated in coastal waters by river systems and coastal populations [29]. Additionally, of greatest concern for northeast Pacific fin whales, the nearshore concentration of marine vessel traffic magnifies the impacts of noise and collisions [31–33].

**Fig 1:**
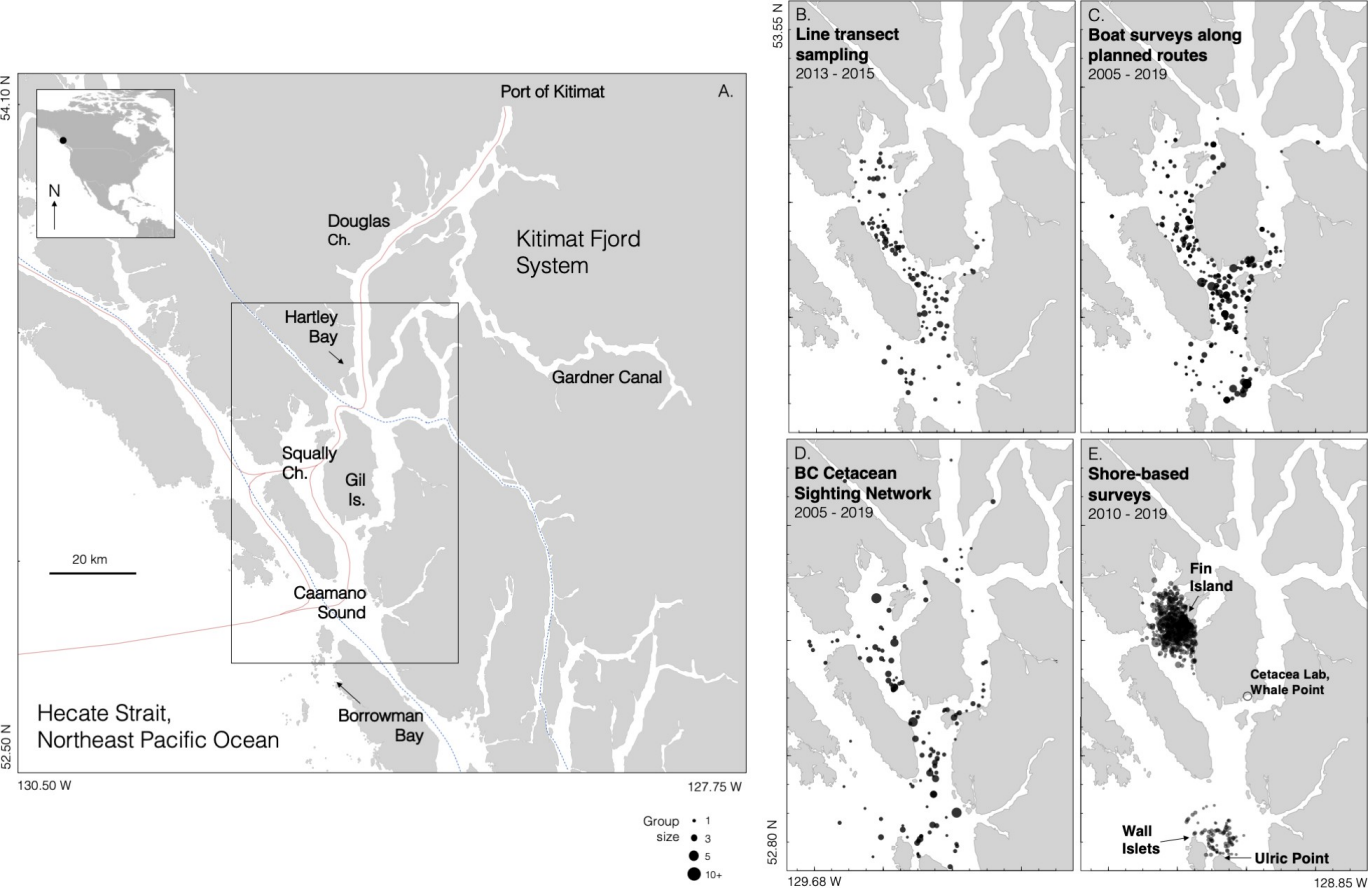
Study area. (A) The Kitimat Fjord System in mainland British Columbia, Canada. Blue dashed line indicates current marine traffic routes; red line indicates new routes that have been proposed for shipping projects based in the port of Kitimat (see text). (B–E) Sightings of fin whales within the Kitimat Fjord System since 1994. Only sightings whose geographic positions are known or estimated are shown here. Survey effort for B-C are provided in S1 Fig in S1 File. In D, sightings data are supplied by the BC Cetacean Sightings Network; these sightings are opportunistic and not connected for effort.

The importance of coastal fin whale subpopulations, as well as their vulnerability, are reflected in the status of fin whales in the Canadian Pacific. This stock remains significantly depleted (> 50%) from regional commercial whaling (1908–1967) [3]. Recent offshore surveys and historical whaling records both indicate that large numbers of fin whales occur above and beyond the continental slope [3, 12]. However, coastal researchers have also identified a putative subpopulation over the continental shelf with approximately 400 individuals who practice strong site fidelity and year-round occupancy to Queen Charlotte Sound and Hecate Strait, with evidently little interchange with the offshore population [34–37]. This offshore-inshore population structure was not formally identified until Nichol et al. [36], and much remains uncertain and unknown regarding these whales (hereafter referred to as a ‘shelf subpopulation’). Compared to the offshore waters of British Columbia, the areas utilized by this shelf subpopulation are host to higher densities of marine debris [38], vessel traffic [39, 40], and anthropogenic noise [39, 41, 42]. The areas that this subpopulation is known to frequent, which range from western Vancouver Island to Dixon Entrance based on current knowledge [36], have been identified as important habitat for the protection of the Canadian Pacific stock [36, 43]. Included within this important shelf habitat is an extreme example of the species’ affinity for nearshore areas: the regular occurrence of a small population within a single mainland inlet, the Kitimat Fjord System (KFS). The KFS is a major physiographic feature of the territories of the Gitga’at and neighboring First Nations within the Great Bear Rainforest (Fig 1A). Although its waters are uncommonly quiet and pristine compared to other coastal areas in northern British Columbia [38, 39, 44], several marine shipping projects are proposed for the area in the coming decade [45, 46]. This study focuses upon fin whale observations in the KFS dating back to 2004. To our knowledge, the KFS is currently the world’s only fjord system that fin whales regularly use, and it is the only place where they are frequently photo-identified from shore. The KFS was also the only Canadian Pacific fjord system in which fin whales were regularly found and killed during commercial whaling [12, 47], pointing to its long-term importance.

Findings regarding fin whales within the KFS have been included in various multi-species studies focused on foraging behavior [48], dive behavior [49], and habitat associations [50]. KFS data have also been combined with other areas in regional studies involving photo-identification [36], whale-borne sensors [36], and passive acoustic monitoring [37]. Several of these regional studies have noted the unusual importance of the KFS to the shelf subpopulation, recommending focused study of its fin whales [35, 36, 43]. Meanwhile, other regional publications, for which data collection [51] occurred just prior to the fin whales’ return to the KFS, have led to the recurrent conclusion that fin whales, since they are presumed absent, face no threats within the KFS and need not be considered in environmental impact assessments within it [38, 39, 41, 42]. A dedicated study is urgently needed to place the present-day role of this inshore habitat within the context of the Canadian Pacific population’s natural history, recovery, and management. Here we investigate historical and contemporary trends in fin whale relative abundance and site use within the KFS. Specifically, our aims were to (1) gather traditional and historical knowledge of fin whales in the KFS prior to the years of modern research; (2) assess recent trends in inland abundance and site fidelity based upon visual surveys and photo-identification mark-recapture; (3) describe spatiotemporal patterns in site use based upon vessel- and shore-based surveys; (4) estimate fin whale spatial density within the fjord system using line-transect sampling; and (5) assess social and demographic patterns in site use based upon photo-identification and photogrammetry from an unmanned aerial system (UAS). Note that, since acoustic data restricted to this area are published elsewhere [52], our focus here is restricted to long-term visual and image-based studies. We pursued these aims by drawing upon historical whaling records, traditional knowledge, citizen science databases, and fifteen years of scientific research from academic, federal, non-governmental, and indigenous scientists.

## Methods

### Data collection

#### Historical accounts

*Early explorers*. The log books of several early explorers that entered the Kitimat Fjord System (KFS) during the late 18th century, including Captain James Colnett (1787), Captain Jacinto Caamaño (1792), Archibald Menzies (1793), and Captain Daniel Pender (1866–68), were examined at the Royal BC Archives and from other sources [53, 54] for indications of fin whale presence and cetacean activity in general (see S1 Appendix for further details).

*Whaling records*. The Historical Whaling Database (HWD, [55]) maintained by the Cetacean Research Program at the Pacific Biological Station, Nanaimo, BC, was queried for all catches of all species that occurred within the KFS in the 20^th^ century. The following information about fin whales killed by whalers in the KFS was sought from the database: location of kills, name of catcher ships that made each kill and the vessels’ originating shore station, the whale’s species, overall body length, sex, fetus presence/absence, fetus sex and length, description of stomach contents, and the number of individuals in the group in which the killed whale was sighted. We also collected records of whale sightings that were documented by some whalers.

*Voluntary sighting reports*. For insights into fin whale activity in and near the KFS during the decades between the end of commercial whaling and the onset of local research, we queried the BC Cetacean Sightings Network (BCCSN), a database of cetacean sightings reported by members of the public since the early 1970s. Data obtained from the BCCSN Network were collected opportunistically with limited knowledge of the temporal or spatial distribution of observer effort. As a result, absence of sightings at any location does not demonstrate absence of cetaceans.

#### Photo-identification

From 2006 to 2019, we collected photographs of the left and right sides of fin whales, including the dorsal fin, under research permit DFO XR 83 2014. The majority of photographs were collected aboard two small research vessels, the *Elemiah* (8m skiff; 2006–2016 and 2019) and the *Bangarang* (12m motorsailer; 2013–2015), whereas a smaller proportion were collected from shore near land-based research stations (Fig 1E). All photographs were collected with digital SLR cameras equipped with telephoto lens (100–400 mm). We noted when individuals occurred in associated groups, defined as coming within two body lengths of each other and coordinating their swimming, diving, and/or ventilation behavior for at least one surfacing, following previous studies [56–59]. Effort data and field observations were managed within a ‘catRlog’ database [60] used to relate the identifications within each encounter to details regarding spatial position, behavioral state, and social associations. For each encounter, all available photographs were reviewed, and the best quality photos of the left and/or right sides were selected for each individual present. Copies of these photos were made, cropped to include just the whale, adjusted for exposure to enhance the visibility of marks, and entered into the database.

#### Line transect sampling

In the summers of 2013–2015, systematic whale surveys were conducted aboard the *Bangarang* with a team of three researchers. For a detailed account of survey design and methodologies, see Keen [61]. Briefly, circuits of the outer and central channels of the Kitimat Fjord System were completed within a target duration of 20 d, during which we visited a grid of oceanographic stations (n = 24), between which we conducted concurrent visual transect surveys (S1B Fig in S1 File). Whale surveys were carried out using line-transect sampling methodology [62]. Bearing and reticle readings using Fujinon 7x50 binoculars, min-max-best group size estimates, and cue behaviors for each sighting were recorded by an observation team from a platform 2 m above sea level. These data were used to geo-locate the positions of detected whales using R package ‘bangarang’ [63], which accounts for earth curvature and horizon obstruction in confined fjord channels.

#### Other boat-based visual surveys

From 2005 to 2019, whale surveys along pre-determined routes in the KFS were carried out by researchers with the Gitga’at Guardian Watchmen Program and North Coast Cetacean Society (NCCS) [64]. Most surveys were conducted at high speeds (25–40 km hr^-1^) and often included the circumnavigation of the fjord system’s central islands in a single day, but the route varied on occasion due to weather and daylight (S1A Fig in S1 File). On both platforms, two to three observers scanned for cues of whale presence such as blows, splashes, flukes, fins, or breaches. Groups were approached slowly in order to estimate group size and record behavior, and an encounter ended once notes were complete or within 30 minutes.

Spatial survey effort was recorded in 2005–2014 by manually recording GPS location from the vessels’ chartplotter. Gitga’at surveys were conducted year-round from small vessels (7m – 9m) and typically began in Hartley Bay (Fig 1). NCCS surveys on the *Elemiah* were launched from the south end of Gil Island in non-winter months during good weather conditions with visibility greater than 3 nautical miles and sea state no greater than Beaufort 3. In 2006, NCCS used an 8m vessel at 12 km hr^−1^. On both platforms, the primary observers remained the same from year to year (authors NR, AD, and CRP on Gitga’at surveys; JW and HM on NCCS surveys).

#### Shore-based visual surveys

Shore-based surveys for fin whales and other marine mammals occurred in 2009–2019 in three study areas (Fig 1). Caamaño Sound, the offshore entrance to the fjord system, was surveyed from a station at Ulric Point on the north tip of Aristazabal Island (52.827 N, 129.275 W; 8.5m above sea level, asl, at 0.0m tide) in 2009–2011 and from the Wall Islets (52.860 N, 129.347 W, >12m asl) in 2014 and 2019. Further into the fjord system, the central waters of the study area were surveyed from Cetacea Lab at Whale Point (53.102 N, 129.179 W, 12m asl) by NCCS in 2011–2018 and the Pacific Whale Society in 2019. Further in, central and northern Squally Channel was surveyed from Fin Island Research Station (53.235 N, 129.368 W; 9.8m asl) in 2017–2019. These platforms were placed 9-15m above mean low tide, and each site practiced a specific version of the same general protocol (details presented below for each site): systematic scans of the viewshed, in which a conscious effort was made to survey the entire visible area evenly, occurred at regular intervals throughout the summer between 6am and 10pm during daylight hours, using magnified optics for improved detection, identification, behavior designation, and group size estimation. Sighting conditions and weather were noted for every scan. All fin whale detections were logged in every scan, even if those particular whales had been detected previously.

In the outer waterway of Caamaño Sound, scans from Ulric Point were conducted by a trained land-based observer (author JP) with occasional volunteer assistance [65]. The year 2009 was treated as a pilot study, though effort was conducted regularly throughout daylight hours. In 2010 and 2011, scheduled scanning took place regularly (15-minute scans separated by 15-minute breaks in 2010; 1-hour scans separated by 30-minute breaks in 2011) throughout daylight hours between late May and late August, and concerted effort was made to track fin whale individuals throughout the day. Survey optics included Pentax 8x40 handheld binoculars with Vortex Skyline ED 20-60x80mm tripod-mounted spotting scope (2009–2010) and 25x power Big Eyes (www.bigeyes.ca; 2011). In 2014, scans from the Wall Islets followed a scanning regime similar to that of Ulric Point in 2010. In 2019, the regime changed to 20-minute scans on the hour for all daylight hours.

In the central study area, fin whales were documented from Whale Point by trained volunteers during regular scans (five minutes every fifteen minutes) from 0700 to 2000 between mid-May and mid-September. Observers were equipped with Nikon 8x40 and 7x50 handheld binoculars as well as a Vortex Skyline ED 20-60x80mm tripod-mounted spotting scope.

Further into the fjord system, fin whales in Squally Channel were documented from Fin Island Research Station by a mixture of professional observers and trained volunteers. Scans of 20-minute duration were conducted hourly from 0700 hours to 2000 hours, with a reduced rate during early afternoon (scans at 1200 hours, 1400 hours and 1600 hours) when lighting conditions were poorest for detecting blows. Scans were conducted using a combination of Big Eyes, spotting scope (Zeiss DiaScope 20-60x85 T*FL; Oberkochen, Germany), Fujinon 8x50 binoculars (Fujifilm Corporation; Minato City, Tokyo, Japan), and naked eye.

Detections from these shore-based stations were geo-located based upon bearing and reticle measurements. Platform height was adjusted according to tide height predictions from nearby tide stations in Borrowman Bay (~5 nmi from Ulric Point and Wall Islets) and Hartley Bay (~10 nmi from Fin Island Research Station). Reticle measurements were converted to distances using RetDistBE, a MS Excel macro (NMML 2012), in the case of Ulric Point data, and custom software [66] in the case of other stations. Scan effort was recorded manually by observers in 2010–2019, and in some years had to be approximated (Ulric Point, 2009–2011; Whale Point, 2013–2019). In 2017–2019 at Fin Island, effort was logged automatically using custom data entry software developed by the author EK.

#### Unmanned Aerial System (UAS)

In 2019, UAS flights were conducted in suitable weather conditions of Beaufort ≤ 2 and with no precipitation or dense fog. The UAS was launched opportunistically from the deck of Fin Island Research Station or launched from the bow of the *Elemiah* by a licensed pilot under research permit DFO XR83 2019. The UAS, a DJI Mavic 2 Pro (www.dji.com), was equipped with 1-Hz LiDAR (Light Detection And Ranging) and GPS data-logger (S2 Fig in S1 File) and flown at a target height of 30m above fin whales (after Dawson et al. 2017; realized range = 30m – 40m). Individual whales were positioned in the center of the frame to minimize measurement error due to elevated levels of lens distortion at the periphery of the frame. The in-built Hasselblad camera was positioned at nadir, a 90° angle pointing directly down at the sea-surface. The pilot aimed to hover in place for multiple seconds over each individual of a group. Behavioral responses to the UAS and/or research vessel, if any, were classified in the field, on a scale adapted from Weinrich [56].

### Analysis

#### Photo-identification catalog development

To develop a historical catalog of the individuals observed within the study area, we followed the matching procedure developed by Falcone et al. [67], Nichol et al. [36], and Keen et al. [60]. Briefly, individuals were identified based upon the shape of the dorsal fin and any available marks or patterns on the sides of the body. Matches were confirmed by at least two experienced analysts based upon combinations of unambiguous features. Left side-right side matches were made using photos of the same whale from within an encounter, and could be made across sightings for whales with highly distinctive dorsal fins (e.g., clearly visible notches and/or disfigurements).

Photos were then scored for identification quality according to a three-tier scale in five categories: photographer angle to whale, photograph exposure, focus, proportion of the body visible, and the distinctiveness of the dorsal fin (generally, 1 = excellent; 2 = adequate; 3 = poor; see Table 1 in Falcone et al. [67]). Once matching was complete, all available photos of each individual were assessed for inclusion in the catalog; to be included, a whale had to have at least one photograph of either side whose quality scores were all above 3. If dorsal distinctiveness was given a score of 1, then the whale may be included in the catalog at the discretion of the supervising analyst (EK).

**Table 1:** Fin whale encounters. Detections of fin whale groups (n = 2,542) in the Kitimat Fjord System, 2005–2020, by the observation platforms involved in this study.

	Observation platform							
	Vessel-based				Shore-based			
Year	*Elemiah*	*Gitga’at*	*Bangarang*	*BCCSN*	*Ulric Pt*.	*Wall Is*.	*Whale Pt*.	*Fin Is*.
2005	-	0	-	1	-	-	-	-
2006	3	0	-	0	-	-	-	-
2007	2	0	-	2	-	-	-	-
2008	1	0	-	5	-	-	-	-
2009	7	4	-	16	52	-	-	-
2010	7	0	-	7	26	-	-	-
2011	17	0	-	3	29	-	80	-
2012	24	5	-	3	-	-	42	-
2013	55	22	43	9	-	-	137	-
2014	36	8	39	7	-	22	18	-
2015	44	5	46	5	-	-	35	-
2016	26	9	-	18	-	-	65	-
2017	0	2	-	10	-	51	122	331
2018	0	4	-	17	-	49	62	54
2019	8	-	-	20	-	129	280	284
**Total**	**230**	**59**	**128**	**127**	107*	**380**	**841**	**670**

**Notes:** (1) Includes “Likely Fin Whales” reported from Ulric Point. Whale Pt and Fin Island only use confirmed FW sightings. (2) Ulric Pt tracked detections throughout the day, while Whale Point and Fin Island did not (i.e., the same individuals could be recounted in several scans throughout the day).

Matching for photographs from 2006–2014 was carried out by scientists of the Cetacean Research Program at Pacific Biological Station (see Nichol et al. [36] for methodological details). These matches formed the basis of our historical catalog for the KFS. Photographs for 2015, 2016 and 2019 were then used to complete the catalog. If a whale was found by at least two experienced analysts to be absent from the historical catalog and was captured in an identification of sufficient quality, it would be assigned a unique ID code and added to the historical catalog.

If an identification was sufficiently adequate to confirm that a whale was unique to all other whales seen in the same year, but too poor to include in the historical catalog, it was assigned the designation “Unique In Season” (UIS). This practice allowed us to track the number of individuals observed in a given year without compromising the quality of the dataset used for interannual site fidelity and abundance estimation analyses. Likewise, we noted identifications of calves or yearlings, whose appearance may be distinct within a field season but whose dorsal shape and degree of marking is liable to change between years.

#### Abundance estimation

Trends in fin whale abundance in the fjord system, including documentation of the species’ return to this habitat, were inferred from effort-corrected encounter rates from boat-based surveys (2005–2015) and mark-recapture analyses based upon photo-identification (2006–2019). To summarize encounter rates on spatial and seasonal scales, sightings and effort (km trackline surveyed) were binned into 24 spatial polygons and 26 bi-weekly periods (14 days each, from 1 Jan to 31 Dec) annually, *sensu* Keen et al. [64].

Mark-recapture abundance estimation followed the methods in Falcone et al. [67] and Nichol et al. [36]. The annual abundance of fin whales using the KFS was estimated in RMark [68] using the POPAN parameterization of the open-population unconditional Jolly-Seber model (POPAN model) [69]. From this model, we also obtained an estimate of the population, i.e., the number of individuals that could or have the potential to use the study area and be available for capture at any point during the study period. The POPAN model also estimates the recapture probability (*p*), the probability of entry into the population (*p*_*ent*_), and apparent survival (*ϕ*), which is the product of true survival and the probability that an individual does not permanently leave the population. Because the number of fin whales who were aware of and liable to use the study area clearly increased over time (see *Results*), we modeled *p*_*ent*_ as a parameter that varied annually. For the other variables of interest, we built models including all combinations of constant and time-varying parameterizations, then compared model performance based upon AICc, selecting as ‘best’ those within 2.0 AICc points of the model with the lowest AICc [70].

#### Site fidelity

We characterized population site fidelity based upon the tendency of fin whales to occupy the study area or to return to it over some period of time [71]. To do so, we used population-level and individual-based metrics of observations, both across years and within field seasons.

*Interannual return*. To characterize interannual site fidelity, we calculated the population’s annual return rate (number of recaptures divided by total captures; two versions of this metric were calculated, in which recaptures were defined either as whales seen in the previous year or as whales seen in any previous year; *sensu* [72, 73], and determined the proportion of study years in which each individual was seen. To determine the impact of dorsal distinctiveness on our analysis of site fidelity and abundance estimation, we compared site fidelity rates across categories of dorsal distinctiveness.

*Seasonal residency*. Population-level residency patterns within a year were examined using Lagged Identification Rates (LIR [74, 75]), which depict the probability that an individual identified on any given day will be re-identified *τ* = 1, 2, 3, …, τ _max_ days hence. We set *τ*_max_ to 230 days within the same year as time 0 (chosen to ensure that identification rates did not wrap from the end of one field season, across the winter months, and into the next year’s field season), used only lags with 5 or more paired identifications to build the LIR curve, and obtained confidence intervals using 100 bootstrap replicates of the data [76].

To evaluate the statistical significance of the observed LIR curve, we compared it to a curve generated by a null model in which whales moved in and out of the study area randomly. To generate these null data, we carried out iterative randomizations of the data stream (after [77]), shuffling the identifications collected in each encounter and recalculating the LIR (n = 1,000 iterations). We compared the observed LIR curve to the 95% confidence interval of the null model (calculated using the 0.025 and 0.975 quantiles of the null distribution of randomized LIR curves), and interpreted any departure of the observed curve from the confidence interval to represent statistically significant patterns in seasonal residency. In this and all permutation tests below, we confirmed that the sample size of the randomization routine was sufficient to achieve a stable p-value.

On an individual basis, seasonal occupancy was characterized using standard indicators of site fidelity, including occurrence (*IO*, proportion of recaptures), permanence (*IT*, the proportion of time spent in the study area) and periodicity (*It*, individual recurrence) (see [78] for details and formulae), as well as the Standardized Site Fidelity Index (SSFI) developed by [78]. The SSFI lends itself to comparison across other feeding and breeding grounds for this species and is robust against irregular survey effort and imperfect detection probability [78]. For each individual, *IO*, *IT*, *It*, and *SSFI* were calculated for each year, then the mean and standard deviation were used to summarize the central tendency and consistency of its occupancy patterns.

#### Distribution & site use

The maximum spatial and temporal range of fin whales within the Kitimat Fjord System was assessed using the full database of geo-located sightings from all research platforms and the BC Cetacean Sightings Network (“certain” report only). Spatial patterns in distribution were inferred from boat-based research surveys (2005–2016) that were effort-corrected and spatially binned as described above, as well as from the results of spatial density estimation from line transect surveys.

Seasonal patterns in occupancy were assessed using monthly counts of fin whales killed by whalers and those reported by citizen scientists in the BCCSN. Effort was not available for these datasets, and they were interpreted with caution given the potential biases inherent to such data. Effort-corrected patterns were resolved on a biweekly timescale from boat-based surveys (fin whales detected per km of trackline surveyed) as well as shore-based surveys (fin whales detected per hour of scan) for years in which effort was well-documented. To aid in cross-platform comparison, encounter rates were scaled by the maximum rate observed in each platform-year so that all seasonal curves peaked at 1.0. These rates were calculated for biweekly bins. To do so, detections and effort from each 14-day bin were combined across years to produce a single encounter rate for each platform.

Average summertime spatial density in the KFS (fin whale groups km^-2^) was estimated using observations from line transect sampling in 2013–2015 and the R package ‘Distance’ as described in Miller et al. [79]. To determine the proportion of fin whales detected within the area covered by our surveys, we modeled a detection function using a set of detections that was truncated according to the 90% quantile of the distances of detections from the survey track line. To model our detection function, half-normal and hazard-rate key functions were tested with and without cosine adjustments, and the best model was selected based upon AIC [70] as well as goodness-of-fit, which was determined by a Cramer-von Mises test (unweighted) in which a non-significant test statistic indicates a plausible model [79, 80]. We did not apply covariate adjustments to our detection function, since line transect surveys only took place in fair weather with a Beaufort Sea State 3 or better, unlimited visibility, and within protected channels without significant swell.

Once the detection function had been modeled, functions within ‘Distance’ were used to estimate fin whale density and expected cluster size for five geographic strata, which were delineated according to the uneven fin whale distributions we observed during fieldwork: the outer waterways of Caamaño Sound (stratum 1) and Estevan Sound (stratum 2), the central passages of Campania Sound (stratum 3) and Squally Channel (stratum 4), and the remaining inner passages of the study area deeper within the fjord system, including the Inside Passage traffic route (stratum 5).

Behavioral patterns and other forms of site use were derived from close observations from both boat- and shore-based research platforms. Behaviors were classified according to the protocols detailed in [61]. Briefly, whales were inferred to be “feeding” when most or all of the following behaviors were observed: travel pattern was circuitous or repetitively back-and-forth; dives were long; surface sequence comprised relatively many breaths during which the animal was uncommonly still at the surface, suggesting recovery from feeding activity at depth; the first breath of a surface sequence was inordinately energetic and subsequent breaths were disproportionately meager; and animal orientation changed before the onset of a dive. Other behavioral states included traveling, milling, resting and sleeping. Traveling was indicated by directed transit with unchanging course, regular and relatively brief dive intervals, and relatively brief surfacings. Milling whales were active but had no directed course and no indication of feeding. Resting whales were either still or moving slowly with relaxed ventilation and uncommon or absent dives. Sleeping whales utterly still and unresponsive to nearby vessels. Historical site use was inferred from stomach content analyses from the commercial whaling data.

#### Sociality

The prevalence of social relationships among catalogued fin whales was characterized using weighted association indices. We selected the Simple Ratio association Index (SRI, [81]) because 1) we lacked calibration data and 2) the biases inherent to the SRI are more predictable than to an alternative such as the Half-Weight Index (after [82, 83]). The stability of social associations was assessed with randomization, in which the individuals within each encounter were shuffled in the dataset, preserving the number of times each individual was seen, and encounter rates for all possible dyads were recorded. This was repeated 1,000 times to develop a null model of random social mixing against which to compare the dyad encounter rates that were actually observed.

#### Demographics

The reproductive activity of the fin whales using the KFS was assessed using the effective per-capita calving rates (i.e., fraction of population represented by calves, as observed on feeding grounds presumably after migration from the breeding area [84–86]), based upon data from platform-years in which calves were carefully recorded.

Photogrammetry from 2019 UAS footage was also used to assess body size, infer demographic patterns, and compare to morphometric data from the whaling records. Stills were extracted from the video footage (PhotoMechanic 5, Camera Bits Inc.) and their timestamps were adjusted according to the video start time. These timestamps were matched to the LiDAR elevation data, in cm, and camera tilt angle, which was used to correct for the horizontal tilt of the UAS induced by headwind. These parameters were used to extract morphometrics using ‘Whalength’ [87] in MATLAB (MathWorks Inc. 2020).

Measurements included total body length (rostrum to fluke notch), body width at 10% increments, rostrum to blow hole, and fluke widths (S3 Fig in S1 File). At least five stills were used from multiple surfacings in each flight to arrive at means and standard deviations of measurements for each individual. When LiDAR data were corrupted due to technical malfunctions, body and fluke widths are presented as percentages of total body length given in pixel length. Dorsal chevron patterns in pigmentation, which are unique for fin whale individuals [88], were used to ensure that we tracked which individuals, if any, were re-measured throughout the season.

## Results

Fifteen years of boat-based observations (2005–2019) and eleven years of shore-based surveys (2009–2019) yielded 2,542 fin whale group detections in the Kitimat Ford System (KFS) since 2005 (Table 1). Boat-based research effort was quantified for 11 of those years (2005–2015), wherein 27,311 km of trackline were surveyed (Table 2). Of that effort, 2,796 km involved line transect sampling, of which nine surveys were completed (mean 22 d and 313 km each) over 102 days of effort between June and September in 2013–2015.

**Table 2:** Search effort. Search effort (km of trackline searched or hours of shore-based scan effort) documented and available for this study.

	Observation platform							
	Vessel-based (km)				Land-based (hr)			
Year	*Elemiah*	*Gitga’at*	*Bangarang*	*BCCSN*	*Ulric Pt*.	*Wall Is*.	*Whale Pt*.	*Fin Is*.
2005	-	1,501	-	-	-	-	-	-
2006	1,136	1,154	-	-	-	-	-	-
2007	811	869	-	-	-	-	-	-
2008	1,355	1,626	-	-	-	-	-	-
2009	673	1,951	-	-	448	-	-	-
2010	828	549	-	-	714	-	-	-
2011	834	911	-	-	774	-	400	-
2012	1,450	2,117	-	-	-	-	444*	-
2013	1,562	2,387	504	-	-	-	444*	-
2014	1,445	1,356	639	-	-	428	444*	-
2015	-	-	1,653	-	-	-	444*	-
2016	-	-	-	-	-	-	444*	-
2017	-	-	-	-	-	-	444*	279
2018	-	-	-	-	-	-	444*	436
2019	-	-	-	-	-	250	444*	623
**Total**	**10,094**	**14,421**	**2,796**	**-**	**1,936**	**678**	3,920*	**1,338**

* Note that Whale Pt scan effort in 2012–2019 is approximated based on field notes.

Shore-based research effort amounted to 6,572 hours of scanning and 1,998 detections (79% of all sightings in this study). The 544 detections remaining (21%) occurred from vessels. Of these, 127 (5% of all detections; 23% of boat-based detections) were contributed by voluntary reports through the BCCSN. Photo-identification, which took place from boat and from shore, began in 2006 and occurred in all years of boat-based research except 2017–2018, yielding 12 years of mark-recapture data in which 672 photo-identifications of 101 individuals were recorded during 257 encounters. Of these identifications, 584 (87%) were of non-calves and were of sufficient quality to receive an ID and be cataloged.

### Historical occupancy

The Gitga’at have known of the whale populations within their traditional territory for generations, though they did not actively hunt them (Elder Ernie Hill, pers. comm. to author JP), and there is no indication of a time prior to whaling that whales were absent from Gitga’at waters. European documentation of abundant whales in the area occurs as early as 1787 [53]. However, neither of these sources distinguishes between species of baleen whale in their histories; specific mention of fin whales did not occur until commercial whaling records in the first half of the 20^th^ century.

#### Commercial whaling

Of the 24,862 whales killed by coastal whaling stations in British Columbia between 1908 and 1967 [89], 129 (0.5%) were taken from within and near the entrance to the KFS. During these years, proposals to establish a shore whaling station in or adjacent to the KFS were filed on three separate occasions (Webb 1988), suggesting that this area was widely acknowledged as a strategic location for whaling efforts. Species taken here included humpback (*Megaptera novaeangliae*), sperm (*Physeter microcephalus*), sei (*B*. *borealis*), blue (*B*. *musculus*), and fin whales. The vast majority (75%) of this catch was fin whales.

**Fig 2:**
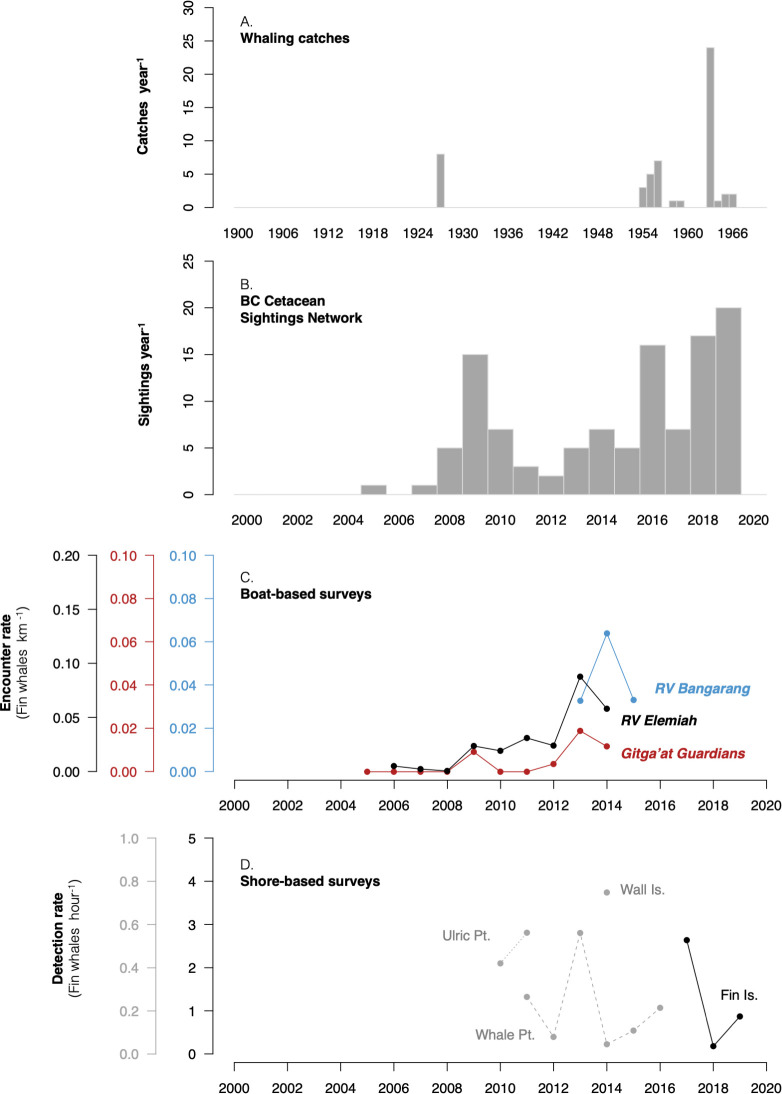
Fin whale detection history. History of documentation of fin whales in the Kitimat Fjord System. (A) Reported kills from the whaling records, not corrected for effort; (B) sightings reported to the BC Cetacean Sightings Network, not corrected for effort; (C) effort-corrected encounter rates (fin whales detected per kilometer of track line surveyed) for three boat-based research platforms, 2004–2015 (note different y-axis scales); (D) effort-corrected detection rates (fin whales detected per hour of scan effort) of fin whales from three shore-based research stations, 2010–2019. Note that shore-based detection rates are for demonstration purposes only; they were not used to assess annual trends in relative abundance.

Between 1927 and 1966, 54 fin whales were killed within the KFS proper. Most catches occurred in Caamaño Sound at the entrance to KFS, but catches occurred on all sides of Gil Island including inland waters to the north (S4 Fig in S1 File). The most catches to occur in a single year came from 1964 (n = 24, 44%; Fig 2). These kills were logged by whalers operating out of Coal Harbour (46 catches, 1954–1966) and Rose Harbour (8 catches, 1927). Based upon itineraries reconstructed from ship logs, the 54 fin whale kills occurred during 87 days of local effort by thirteen different whaling ships, though other efforts and vessels may have gone undocumented. Rose Harbour documented kill locations only in 1924–1928, meaning other fin whales may have been taken from the KFS in other years.

In addition to the time series of whales killed within the KFS (Fig 2), visual sightings were documented by whalers from 1963 to 1967. In that time, fin whales accounted for 94% of the 273 detections logged near the KFS. This prevalence may be indicative of relative abundance, but it is possible that whalers were selectively recording sightings of more desirable species. See S1 Appendix for a detailed history of early exploration and whaling activities within the KFS.

### Recent trends in abundance

For the years between the end of commercial whaling in BC (1967) and 2005, we were unable to find any confirmed or high-confidence records of fin whale occurrence within the KFS despite the facts (1) that citizen contributions to the BC Cetacean Sightings Network (BCCSN) began in the mid-1970s [90], and (2) that Gitga’at mariners were actively using the waters throughout their territory on a near-daily basis during those years. Based on the authors’ extensive conversations with Gitga’at elders, there is no memory of fin whales in Gitga’at waters between the end of whaling and the mid-2000’s. The earliest BCCSN records of fin whales within 50km of the entrance to the KFS at Caamaño Sound comes from 1994–1995, though these records have a confidence classification of Probable rather than Certain (S5, S6 Figs in S1 File). The first Certain report within this range occurred in 2002, which was the same year in which a Probable sighting occurred within the KFS proper. The first Certain report from within the KFS did not occur until 2005, and the first sighting by a research group did not occur until 2006 (authors JW & HM), two years after the onset of formal research and four years after the researchers became year-round residents in Gitga’at territory and initiated informal surveys.

In each year since 2006, fin whales were observed within the KFS by citizen contributors as well as cetacean research groups (Fig 2). Effort-corrected encounter rates from boat-based survey platforms indicated an accelerating increase in local abundance from 2005 to 2014, after which both boat- and shore-based platforms observed wide fluctuations in year-to-year abundance (Fig 2).

During years of photo-identification effort (2006–2016, 2019), the annual number of unique whales observed per year ranged from 1 to 41 (2008 and 2013, respectively) (Table 3). The rate of new identifications began slow (20 individuals identified in the first five years, 2006–2010), then increased substantially until 2015. The discovery of new individuals slowed in recent years but has not stopped (S7 Fig in S1 File), indicating that new individuals continue to be recruited to this inland population.

**Table 3:** Photo-identification record. Capture history of photo-identified fin whale individuals in the Kitimat Fjord System, 2006–2019 (all distinctiveness categories).

Year	Identifications				Recaptures					
	Total	Unique	New	Catalog	Within season		Prior year		Any prior year	
					*n*	*%*	*n*	*%*	*n*	*%*
2006	6	5	5	5	1	17%	0	0%	0	0%
2007	2	2	2	7	0	0%	0	0%	0	0%
2008	1	1	1	8	0	0%	0	0%	0	0%
2009	14	13	11	19	1	7%	1	8%	2	15%
2010	20	5	3	22	15	75%	1	20%	2	40%
2011	36	17	13	35	19	53%	1	6%	4	24%
2012	42	15	3	38	27	64%	11	73%	12	80%
2013	191	41	25	63	150	79%	12	29%	16	39%
2014	89	25	6	69	64	72%	18	72%	19	76%
2015	91	26	6	75	65	71%	13	50%	20	77%
2016	73	25	6	81	48	66%	12	48%	19	76%
2017	-	-	-	-	-	-	-	-	-	-
2018	-	-	-	-	-	-	-	-	-	-
2019	19	13	2	83	6	32%	6	46%	11	85%
**Mean** since 2010	70	22	8	-	55	69%	10	43%	13	62%
**SD** since 2010	57	11	8	-	46	9%	6	24%	7	24%

The 584 photo-identifications catalogued in this study represented 83 unique individuals (mean 7 identifications per individual throughout the study). Of these individuals, 56 (67%) were encountered more than once during the study, 43 (52%) were encountered three or more times, 18 (22%) were encountered more than 10 times, and one was encountered on 37 occasions (S1 Table in S1 File).

**Fig 3:**
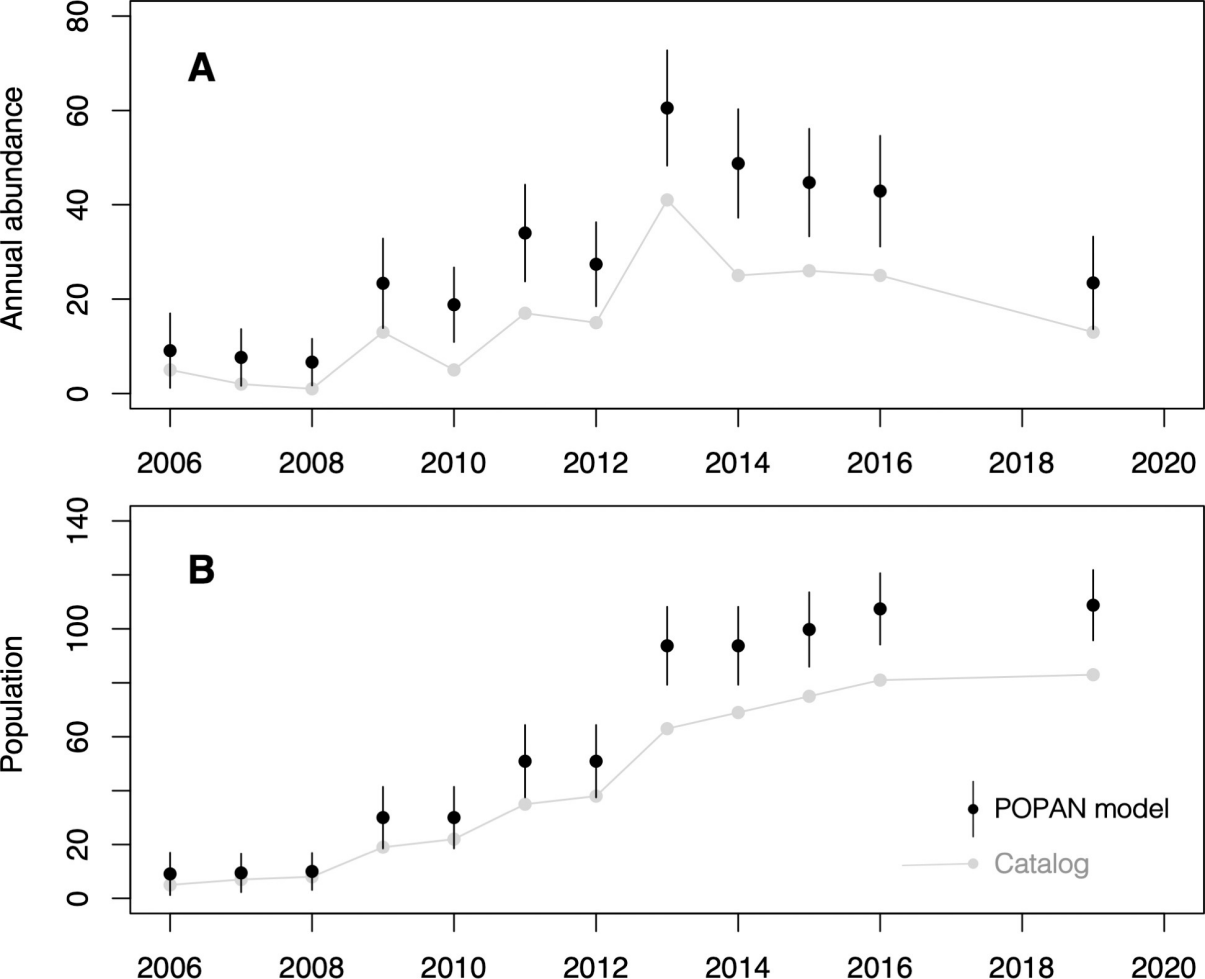
Population trends. Results of photo-identification mark-recapture abundance estimation using POPAN models (black, with 95% confidence intervals), displayed alongside catalog size (grey). A. Annual abundance; B. Population.

The highest-performing mark-recapture POPAN model (S2 Table in S1 File) estimated that the number of fin whales using the fjord system on an annual basis increased from 2006 to 2013 (61 whales in 2013, 95% CI = 48–73), after which annual abundance stabilized then declined between 2016 and 2019 (Fig 3). Since 2010, mean annual population was 38 individuals (SD = 14, 95% CI = 27–48). This model also estimates the population of fin whales using this fjord system to have been 109 whales (95% CI = 96–122) as of 2019 (Fig 3). This was 27% (95% CI = 20% - 34%) of the population estimate in [36] for the coastal waters of Queen Charlotte Sound and Hecate Strait.

### Site fidelity

#### Interannual site fidelity

**Fig 4:**
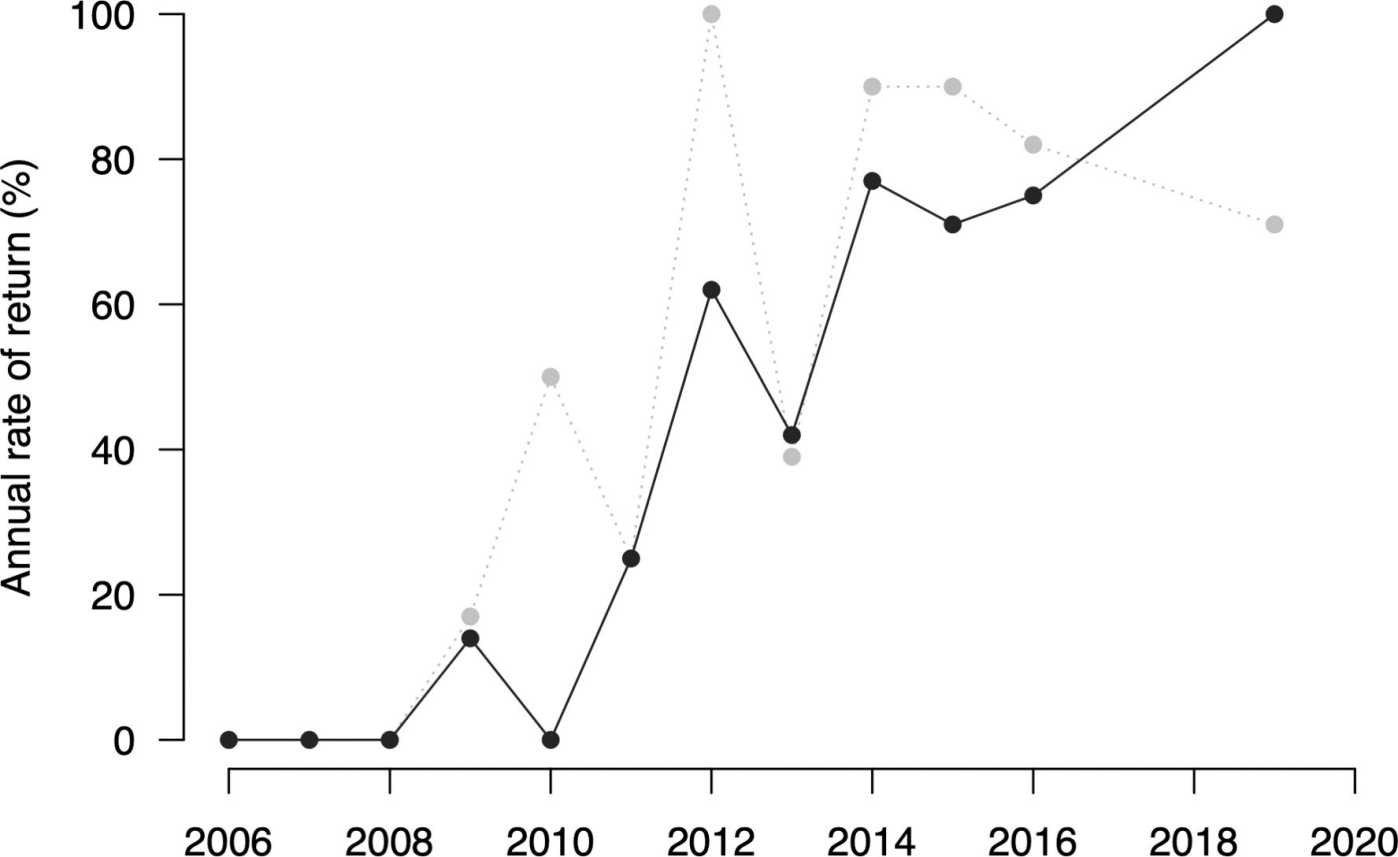
Site fidelity. Annual rate of return (recaptures from any previous year) for whales with dorsal distinctiveness score of 2–3 (black, solid line) and 1 (grey, dashed line). Distinctiveness scores (1 = Extremely distinctive, 2 = adequately distinctive; 3 = indistinct and difficult to identify) are displayed separately to assess for the influence of distinctiveness on perceived rates of return (see main text for further details).

Site fidelity was not observed for the first three years of our study (0% annual rate of return, though this may be biased low due to low annual abundance in those years). As measured, site fidelity began to increase after 2009 (Table 3 and Fig 4). In the final half of our study (2012–2016 and 2019), mean annual return was 70% (year-to-year) to 72% (any previous year), with the greatest return rates occurring in 2013 and 2019, respectively (Table 3). There was some variation in annual return across fin distinctiveness categories (means ranged from 55% for moderately distinctive whales to 79% for extremely distinctive whales) (S3 Table in S1 File), but this effect was not significant (Kruskal-Wallis rank sum tests; p = 0.30–0.53).

Of the 83 individuals in our catalog, 37 (40%) were seen in more than one year, 12 (13%) were seen in 5 or more years, and one was seen in 9 years (75% of study years) (S1 Table in S1 File). On an individual basis, the mean annual return was 19% (SD = 15%, min = 8%, max = 75%) for the entire catalog. When filtered to individuals seen in more than one year, the mean individual-level annual return increases to 32% (SD = 15%, min = 17%, max = 75%) (S4 Table in S1 File).

#### Seasonal residency

Within-season recapture rates were initially low (0% - 17% from 2006–2009), then became high for the remainder of our study (2010–2019 mean = 64%, SD = 15%, min = 32%, max = 79%) (Table 3). Lagged Identification Rates (LIR) indicate that fin whales tended to remain in the study area for only a few days at a time, but intermittently returned to the area throughout the season (S8 Fig in S1 File). Some re-sights occurred up to 80–110 d after initial observation. Permutation tests of the LIR curve indicated that, for time periods greater than 5 d, identification rates were consistent with random, repeated movements out of and back into the study area (S8 Fig in S1 File). Across years, the mean documented occupancy within the fjord system (days between first and last observation, allowing for departure and return) was 18 d (SD = 23 d, min = 1 d, max = 110 d) (S4 Table in S1 File).

### Distribution & site use

#### Spatial distribution

**Fig 5:**
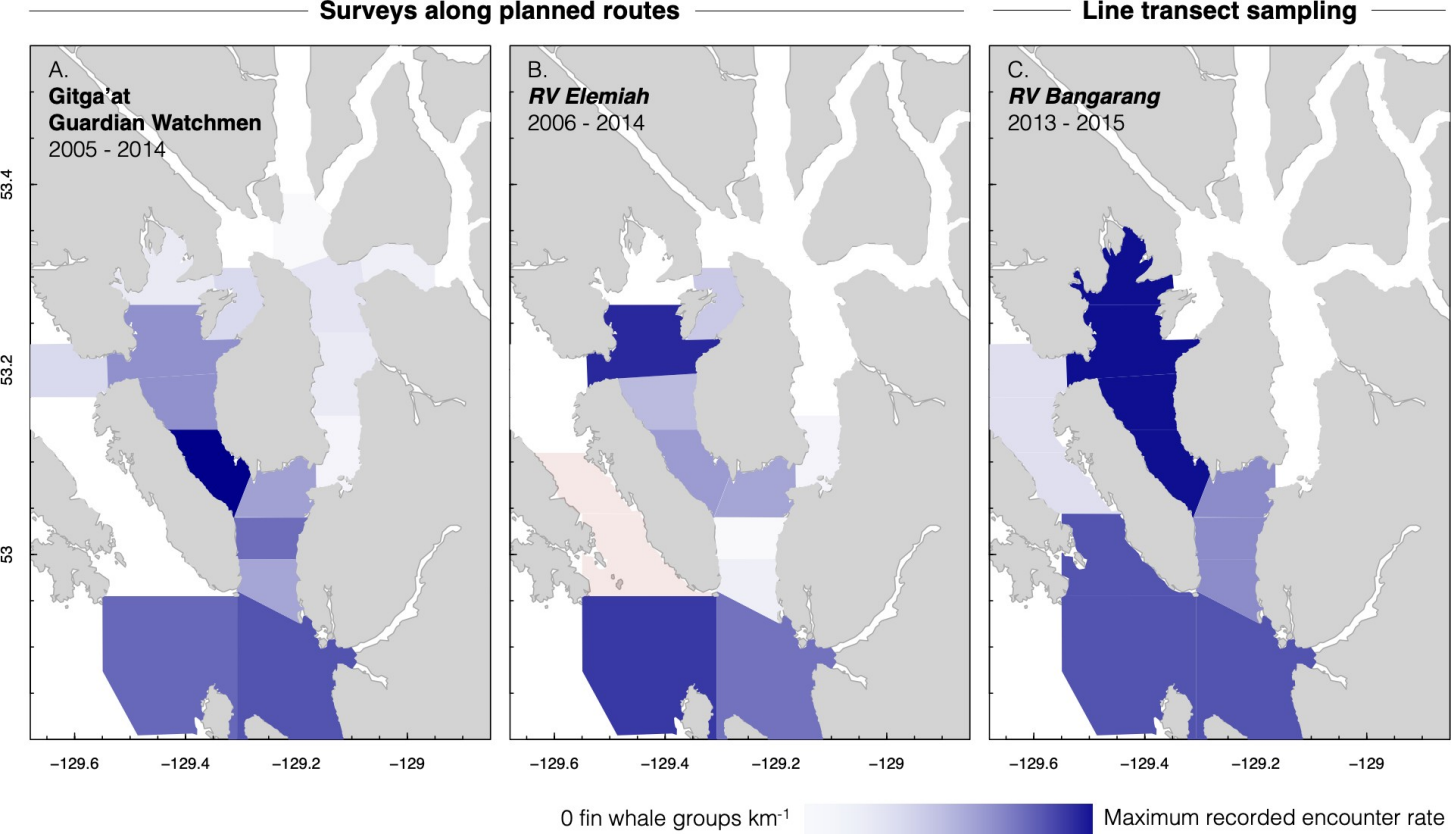
Fin whale distribution. Effort-corrected encounter rates of fin whales in the Kitimat Fjord System, for (A–B) surveys along planned routes, 2005–2014 (see S1 Fig in S1 File for effort), and (C) line transect sampling, 2013–2015 (see S1 Fig in S1 File for effort). In A and B, rates are binned spatially using the blocking from Keen et al. (2017). In C, binning was based upon the strata used in density estimation (S6 Table in S1 File). The color scale in each map is scaled by the maximum observed by the respective research platform. Pink coloration indicates no survey effort.

Boat-based platforms encountered fin whales in most outer and central waterways of the KFS, with almost no records to the north and east of Gil Island (Fig 1B–1E). Effort-corrected boat-based surveys (2005–2015) documented the highest fin whale encounter rates in Squally Channel (central-inner waterways of the KFS, Fig 1A) and Caamaño Sound (outer entrance to the KFS) (Fig 5). Line transect analyses supported this distribution (next subsection).

#### Spatial density

We logged 45 fin whale detections during line transect sampling in 2013–2015 (Fig 1B). Applying a 90% quantile truncation distance of 2.13 km, we modeled the detection function using 38 sightings with valid trackline distance estimates. The best-fit model was a half-normal detection function without cosine adjustments (S5 Table in S1 File and S9 Fig in S1 File). Average probability of detection within the truncation distance was 0.507 (SE = 0.068, CV = 0.135). Based upon this detection function, the total area searched using line transect methods was 9,902 km^2^ (S6 Table in S1 File).

Fin whale density was highest in the central waterways of Squally Channel, with an average of 0.018 whale clusters km^-2^ (95% CI = 0.011–0.031). The next highest density occurred in the outer channel of Caamaño Sound (stratum 1; mean = 0.015 whale clusters km^-2^, 95% CI = 0.006–0.034) (S6 Table in S1 File). Fin whales were only observed once in Estevan Sound during formal survey effort, and no detections occurred within the inner channels (stratum 5, S6 Table in S1 File) during line transect sampling. At these densities, the expected number of fin whale groups within the fjord system at any given time during the June–September 2013–2015 was 9 groups (95% CI = 6–15) of 18 total individuals (11–28) (S6 Table in S1 File).

#### Seasonal distribution

**Fig 6:**
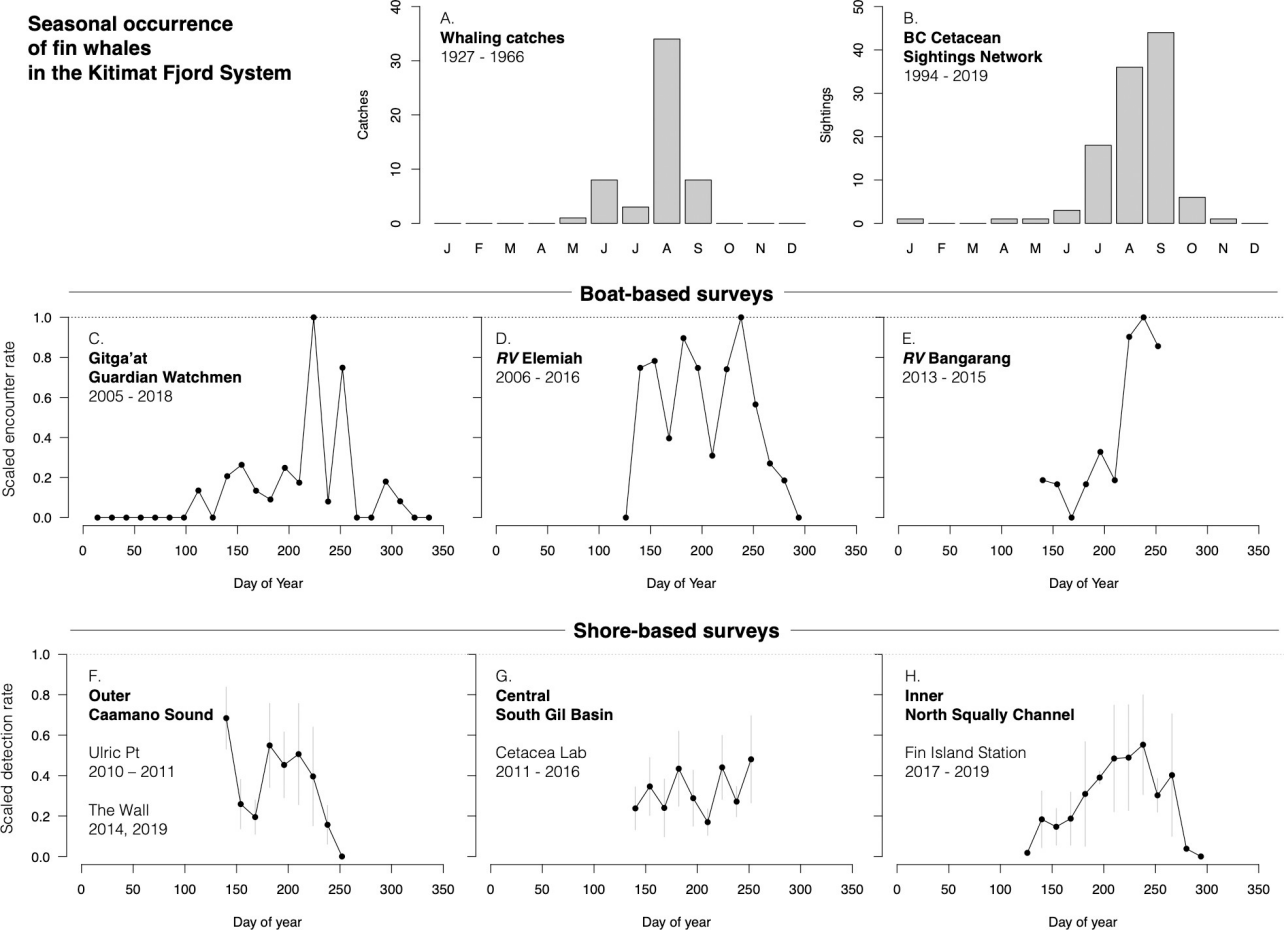
Fin whale seasonality. Seasonality of fin whale occurrence in the Kitimat Fjord System. (A) Monthly distribution of kills during local whaling, 1927–1966, not corrected for effort. (B) Monthly distribution of sightings reported to the BC Cetacean Sightings Network, 1994–2019 (“certain” reports only), not corrected for effort. (C–E) Effort-corrected encounter rates (whales detected per km transited) in two-week calendar bins, scaled by the maximum observed during surveys along planned sourtes by the Gitga’at Guardian Watchmen (2005–2018) and aboard RV Elemiah (2006–2016) and during line transect sampling aboard RV Bangarang (2013–2015). (F–H) Effort-corrected detection rates from shore-based observation platforms in the fjord system’s outer waters of Caamano Sound (2010, 2011, 2014, 2019), central waters in south Gil Basin (2011–2016), and Squally Channel further inland (2017–2019). Shore-based detection rates (whales detected per hour of scan effort) were scaled by the maximum observed in each year then averaged across years for each two-week calendar bin. Grey vertical bars indicate standard error about the mean (black dot).

During commercial whaling in the 20^th^ century, fin whales were killed in the KFS in May–September, with most catches occurring in the month of August (n = 34, 63% of all KFS catches; Fig 6A), though this may have been a function of undocumented biases in effort or record keeping. Since 2005, fin whales were observed in the KFS in April–November (Fig 6B–6E) as well as in January (Fig 6B). The photo-identification record for fin whales in the KFS spans 18 March to 27 October and the mean date of first identification each year is day 210 (late July). The mean date of final identification is 277 (early October; S4 Table in S1 File). Given that visual observations that occurred in November to February were typically impacted by poor weather, these observations likely underestimated the seasonal extent of fin whale presence in the KFS.

Effort-corrected boat-based surveys from various platforms agreed that a seasonal peak in fin whale abundance occurs near day 250 (early September; Fig 6C–6E), and this same peak was seen in Squally Channel from Fin Island Research Station (Fig 6H). However, most boat- and shore-based platforms found other periods of regular fin whale presence, such as days 150 (late May) and 180–200 (late July), and peaks varied from year to year from land-based platforms (S10 Fig in S1 File). Encounter rates from several platforms oscillated strongly throughout the field season, which was consistent with LIR results (S8 Fig in S1 File) and may have been indicative of repeat entry-exit events of several fin whale groups throughout the summer months (Fig 6C–6H and S10 Fig in S1 File).

Though our number of annual samples was low, land-based platforms provided some evidence of geographic differences in the seasonal timing of peak abundance, in which peaks occur later in the year further into the fjord system (S11 Fig in S1 File). However, this pattern was confounded with a second pattern, in which the date of peak detection rate increased from 2010 to 2019 (n = 10, p = 0.005, r^2^ = 0.052; S11 Fig in S1 File). Since not all shore-based platforms were active in all years, we were not able to disentangle these patterns.

*Proximity to shore*. While most fin whale encounters occurred near the centers of KFS waterways (Fig 1), of special note were the many encounters that occurred very close to shore. Fin whales passed within 50 m of each shore-based station at least once throughout our study, dozens of photo-identifications were collected from the rocks when fin whales approached within 20m - 150m, and, in 2019, 65% of morphometric data points were collected during flights launched from shore. During these nearshore events, whales were observed engaged in directed travel, slow transit suggestive of resting behavior, and high-energy movements suggestive of feeding.

#### Behavior

**Fig 7:**
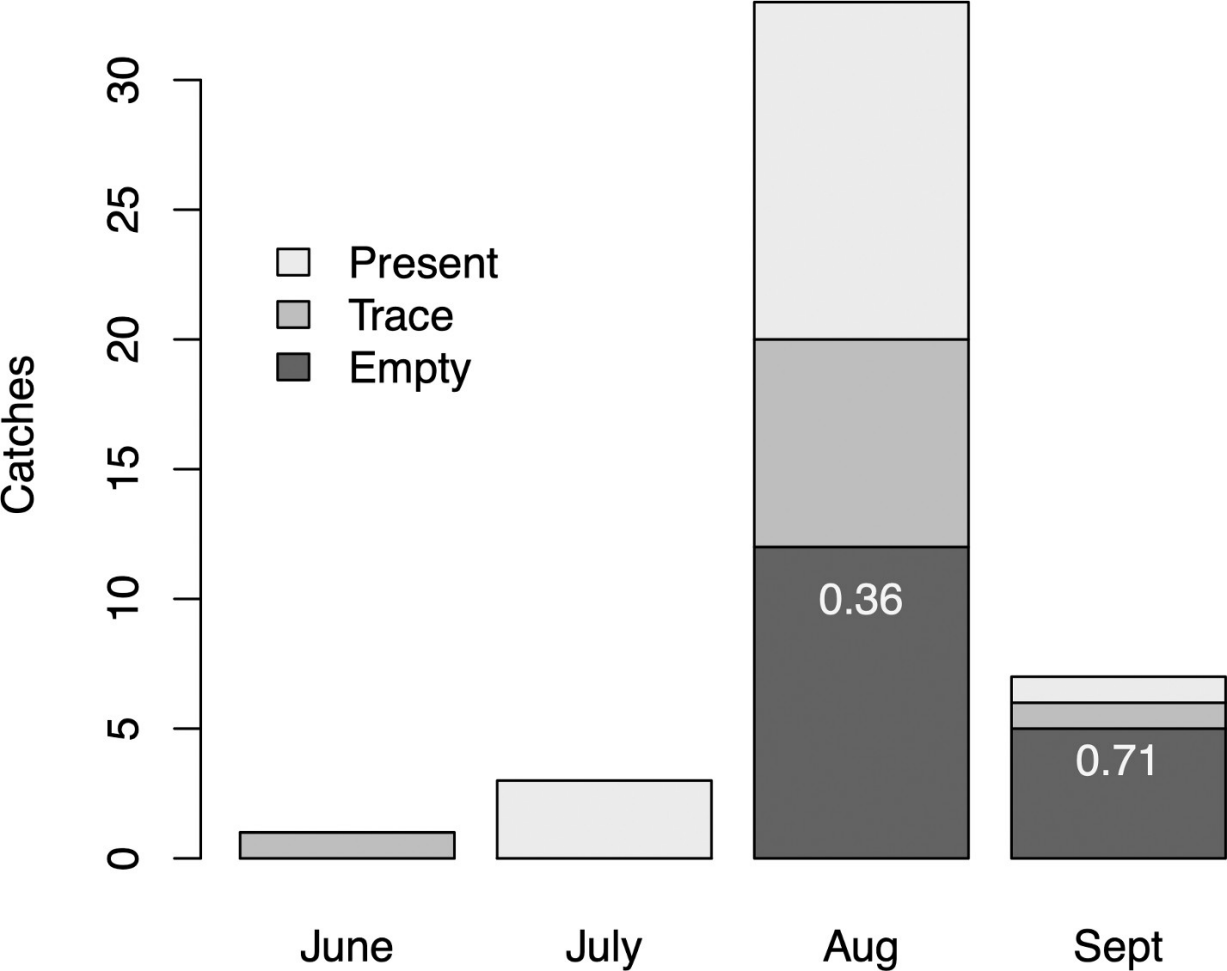
Stomach contents. Presence of euphausiids in the stomachs of fin whales caught by Coal Harbour whalers within the KFS, 1954–1966 (n = 44 inspected stomachs).

Though stomach contents can be difficult to interpret [91], those documented during commercial whaling operations indicated that fin whales were feeding in or near the KFS during early summer in those years. Stomach contents were examined for 44 whales (81% of catches). Of these, 17 stomachs (37%) were empty, 17 had trace amounts of feed, and 10 (23%) contained substantial amounts of prey remains, and the only type of prey noted were euphausiids. No stomachs from June or July were empty (n = 4), 36% of stomachs from August (n = 33) were empty, and 71% of stomachs from September were empty (n = 7) (Fig 7). Curiously, this decline in stomach contents during August and September coincided with the historical peak in catch numbers and the current peak in abundance that we have observed in recent years.

Recent boat- and shore-based behavioral records concurred that fin whales used the KFS during the summer in part to feed. Observations from line transect sampling and associated focal follows aboard *RV Bangarang* have been reported elsewhere [49, 61]. Briefly, 65% of encountered fin whales appeared to be actively engaged in feeding, based upon travel pattern, dive behavior, and associated acoustic backscatter [48]. Side lunge feeds at the surface were regularly observed from the *Bangarang* (author EK) as well as from Ulric Point (authors JP and EK).

Other behaviors, unrelated to feeding, were also observed. These include sleeping, relaxed slow travel, rapid fast travel, as well as social behavior including pair-porpoising, where two individuals suddenly split from their group and began porpoising (i.e, breaching regularly during rapid travel) for a period of several minutes, then slowing down and milling (authors JP and EK on separate occasions). Fin whales were also seen interacting closely with northern resident killer whales (*Orcinus orca*) (JP, JW, HM, on separate occasions) as well as Dall’s porpoises (*Phocoenoides dalli*), who repeatedly rode the bow-wave created by a fin whale who was surfacing vigorously during a feeding session (EK, 2014).

#### Sociality

The mean and median fin whale group size across all observation platforms was 2 whales (S7 Table, S12 Fig in S1 File). Of all groups encountered, 45% were of solo whales, 31% were of pairs, 97% had five or fewer whales, and the maximum size was 12. This matched the mean size observed during line transect sampling (1.9 whales, SE = 0.17, CV = 0.09), in which group size appeared to be slightly higher in the central inner channels (Squally Channel mean = 2.04, SE = 0.17, CV = 0.08) than in outer channels (Caamaño Sound mean = 1.86, SE = 0.34, CV = 0.18), though this difference fell short of significance (S6 Table in S1 File). In general, group sizes documented from vessels were slightly larger than those documented from shore (Kruskal-Wallis, chi-sqp = 9.05, df = 1, p = 0.003; S7 Table in S1 File), which may indicate that individuals are more often missed from the distant vantage of a shore-based platform.

Of the 83 fin whales that we tracked in our photo-identification catalog, 78 (94%) were observed in association with at least one other individual at some point in our study (S8 Table in S1 File). Of the 3,403 possible dyadic associations, 331 were observed (10%). We observed certain dyads more than once; 69 dyads of 41 individuals were encountered twice or more, 7 dyads (10 individuals) were seen four times or more, and 1 dyad was encountered on 13 occasions. There were 36 dyads (24 individuals) observed in multiple years, and two dyads (3 individuals) were observed together in four separate years. However, permutation tests indicated that the stability of these associations was not necessarily greater than would be expected from random association-dissociation processes. Under the null model of random interactions, the probability of observing the same dyad in four years was near-certain (p = 0.99), while the probability of observing the same dyad in 13 encounters was borderline-significant (p = 0.076).

#### Demographic patterns

Effective per-capita calving rates were estimated from 2010 to 2019, each year from at least two of six different research platforms (Table 4). A mean rate of 0.041 (sd = 0.030, min = 0.0024, max = 0.0938) was observed across these platforms. This rate was similar to the proportion of known mothers within our historical catalog (0.032). When sorting land-based calving rates geographically into ‘outer’ samples (Caamaño Sound), ‘central’ samples (South of Gil Island), and ‘inner’ samples (Squally Channel), we found inconclusive evidence of higher calving rates in the outer channels (S13 Fig in S1 File).

**Table 4:** Cow-calf detections. Effective per-capita fin whale calving rate for the platform-years in which cow-calf pairs were documented.

				Annual proportion of individuals noted as calves		
Research platform	Years with calves noted	Total individuals	Total calves	*Mean*	*SD*	*Range*
Bangarang	3	144	4	0.0279	0.0102	0.0182, 0.038
Ulric Point	3	248	12	0.0517	0.0583	0, 0.1149
Wall Islets ‘14	1	32	3	0.0938	-	-
Whale Point	6	587	18	0.0378	0.0228	0.0103, 0.0645
Gitga’at	8	113	5	0.0323	0.0480	0, 0.1250
Fin Island	3	1,357	5	0.0024	0.0028	0, 0.0054

**Fig 8:**
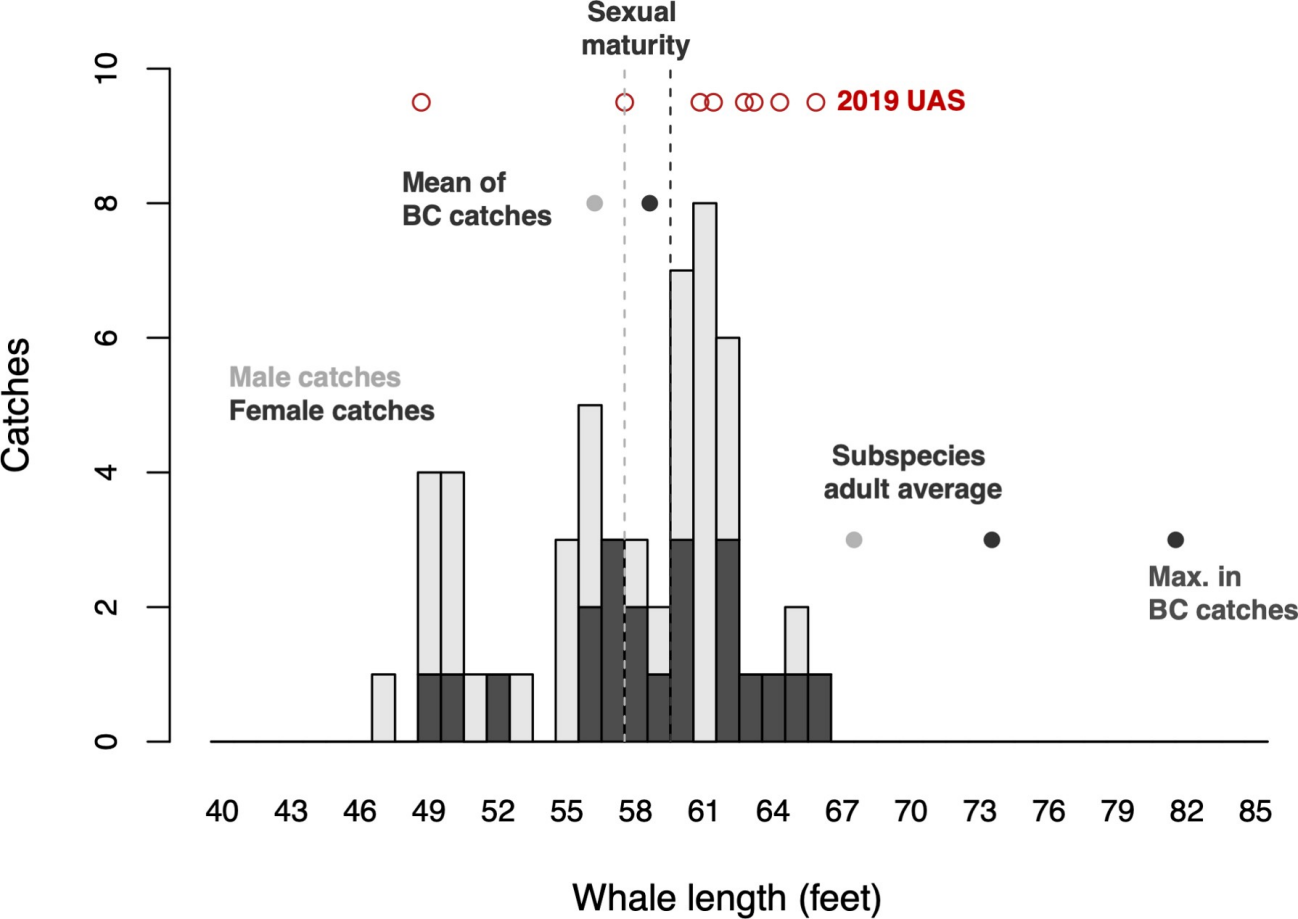
Historical comparison of body dimensions. Lengths of fin whales killed within the Kitimat Fjord System (n = 54) between 1927 and 1966 (histogram; light grey = males; dark grey = females) compared to the whales in the KFS measured remotely by UAS in 2019 (red dots) and published lengths (left to right: mean lengths in BC whaling database (dots, Gregr et al. 2000); length at sexual maturity (dashed lines, Mizroch et al. 1984); average adult length for the North Pacific subspecies (dots, Aguilar 2009); and maximum recorded length in BC whaling database (Gregr et al. 2000).

Footage from the UAS allowed for morphometric measurements of 8 fin whale individuals in 2019 (Table 5; measurements are also provided in feet here to facilitate comparison to [89]). Mean body measurements ranged from 14.98 m (sd 0.17 m; 49 ft, sd 0.56 ft), a juvenile or subadult who was travelling with three larger whales, to 20.22 m (sd 0.44; 66 ft, sd 1.44 ft). Mean fluke width for all individuals was 20.81% of total body length. The lengths measured by UAS in 2019 were within the range, but slightly to the right of the distribution, of lengths recorded during 20^th^ century whaling in the KFS (Fig 8).

**Table 5:** Body dimensions. Mean (standard deviation) measurements (in m) of individual fin whale body lengths and widths based on the number (N) of stills used from 2019 UAS footage recorded at a given elevation (m). See S3 Fig in S1 File for diagram of measurement protocol.

	Body length	Width				Stills	
Individual		*10%*	*30%*	*60%*	*Fluke*	*N*	*Elevation*
20190810 2A*	NA	8.94% (0.59%)	13.26% (0.84%)	9.53% (0.42%)	22.20% (0.21%)	7	NA
20190907 1A	17.68 (0.23)	1.53 (0.03)	2.51 (0.11)	1.81 (0.01)	NA	2	29.90 (0.06)
20190908 1A	19.74 (0.21)	1.73 (0.10)	NA	1.68 (0.04)	3.85 (0.05)	5	35.36 (0.29)
20190911 1A	18.68 (0.25)	1.71 (0.14)	2.60 (0.07)	1.94 (0.08)	4.04 (0.07)	9	42.24 (6.44)
20190911 1B	20.22 (0.44)	1.72 (0.06)	2.56 (0.10)	1.70 (0.08)	4.03 (0.06)	17	43.28 (1.98)
20190911 1C	18.86 (0.37)	1.62 (0.08)	2.63 (0.08)	1.97 (0.04)	3.71 (0.00)	6	45.71 (0.08)
20190911 1D	14.98 (0.17)	1.29 (0.10)	2.02 (0.11)	1.41 (0.08)	3.13 (0.10)	8	40.10 (7.64)
20190919 1A	19.27 (0.45)	1.81 (0.07)	2.57 (0.10)	1.74 (0.21)	4.41 (0.08)	15	40.24 (0.75)
20190919 1B	19.40 (0.33)	1.52 (0.05)	NA	1.63 (0.04)	3.84 (0.11)	5	40.24 (0.14)

* = Case where LiDAR was unavailable, so widths are provided as a percentage of body length.

KFS catches were comprised of 33 males (61%) and 21 females (39%), with overall lengths ranging from 14.33 m to 20.12 m (mean 17.56 m, sd 1.49 m) (47 ft to 66 ft; mean 57.6 ft, sd 4.9 ft). Males were significantly shorter in length (mean 17.3 m, 56.9 ft; sd 1.5 m, 5.0 ft; max 19.8 m, 65 ft) than females (mean 17.9 m, 58.7 ft; sd 1.4 m, 4.6 ft; max 20.1 m, 66 ft) (Kruskal-Wallis rank sum test, chisq = 5.8198, df = 1, p = 0.016). These lengths were similar to the average catch size throughout BC (males = 17.3 m, 56.7 ft; females = 59.1 ft, 18.01 m [89]); they straddled the length at sexual maturity according to whaling data throughout the North Pacific (males = 17.7 m, 58 ft; females = 18.2 m, 60 ft [92]); and they were more than ten feet less than the subspecies average (males = 20.7 m, 68 ft; females = 22.6 m, 74 ft [88]) and more than twenty feet less than the maximum measured length reported from BC catch records (25 m, 82 ft [89]) (Fig 8).

None of the 20 females that were inspected by whalers was carrying a fetus. For the nine females whose mammary glands were inspected, seven were non-parous (i.e., sexually immature or ovulating but not yet pregnant). The lengths of these individuals ranged from 56 to 60 feet. The mammary glands of the remaining two (62–64 feet in length) were involuted, indicating that they had given birth previously.

Altogether, these findings indicated that, notwithstanding the unusually small and young fin whales that occurred throughout BC during the decades of commercial whaling, fin whales of the KFS did not appear to be demographically different from those occurring in other sites visited by BC whalers.

## Discussion

Through fifteen years of study, we found that fin whales have increased within a Canadian Pacific fjord system and have established a seasonally resident population in its intracoastal waters. Traditional knowledge and archival databases lead us to frame these events as the repopulation of an historically important area. Together, these records suggest that the practice of using the Kitimat Fjord System (KFS) by the region’s fin whale population may have ended as a result of whaling in the early- and mid-20^th^ century. Following decades of apparent absence in the wake of that hunt, fin whales now use the KFS regularly once again. These fin whales in the waterways of the Great Bear Rainforest serve as an extreme example of the species’ uncommon practice of establishing residency within coastal areas and semi-enclosed seas.

### Site fidelity and residency

The process of repopulating the KFS, which began in 2005–2006, included a steady increase in the number of fin whales using the area (Fig 3B) as well as an annual rise in local site fidelity (Fig 4). The degree of site fidelity to the KFS has risen even as the number of fin whales using the fjord system each year stabilized and declined (Fig 3A), indicating that the KFS population, while open, is increasingly composed of seasonal residents. The site fidelity rates we have observed in recent years (46–72% year-to-year rate of return, Table 3) are, to our knowledge, the highest recorded for any fin whale site (e.g., [93–95]).

We observed fin whales in the KFS in most months of the year, with regular detections in June—October and the highest encounter rates in late August and early September. These visual observations align well with acoustic detections from hydrophones in the central KFS (Squally Channel), though acoustic surveys have verified occasional fin whale presence from December to March [52]. Within this general pattern, however, the seasonal abundance curve varied across years, and it often featured multiple peaks (Fig 6, S10 Fig in S1 File). These findings, combined with Lagged Identification Rates that remained low but non-zero over a period of months (S8 Fig in S1 File), indicate that fin whales arrive and depart from the KFS repeatedly throughout the summer and fall. This conclusion aligns with our experiences during the fieldwork, as well as with findings from [36], in which fin whales tagged within the KFS were found to leave and return to the KFS on a regular basis.

### Repopulation dynamics

#### Removal

Though we are unable to prove the absence of fin whales from the KFS in the decades between the end of whaling (1967) and the first confirmed detection in 2006, the lack of sightings by contributors to BCCSN and particularly Gitga’at residents allow us to conclude that fin whales were so rare during those years that they were effectively absent. When framed within the historical context of whaling, the repopulation of this fjord system could be interpreted in several ways. Coastal habitats were easily accessed and therefore thoroughly hunted by commercial whalers [96]. As with many other severely depleted taxa, the fin whale’s spatial range and repertoire of habitat use strategies likely contracted, along with their numbers, during the age of commercial whaling [97]. The long absence of fin whales could be explained as a matter of (1) ignorance, if whaling removed all individuals who might be aware of viable habitat within the KFS; (2) avoidance, if the whales that survived whalers in the KFS regarded the area as dangerous; (3) preference, if the depletion of whale populations freed up resources in habitats that were more productive or otherwise optimal; or (4) some combination of these factors.

#### Return

For each of these explanations of the fin whales’ apparent absence from the KFS, their increase beginning in 2005–2006 could be explained variously as (1) a natural result of a reduced population’s expansion into previously important habitat; (2) an accidental discovery unrelated to trends in population growth or resource availability; (3) some change in the relative quality of the KFS compared to alternative habitats, induced either by improvements within the KFS or through the deterioration of habitat elsewhere, perhaps through increased competition, reduced food availability, and/or degraded attributes of the habitat unrelated to prey; or (4) again, some combination of these processes. All of these hypotheses strike us as possible. In fact, it is probable that the relative quality of the KFS has increased in recent decades, given that oceanic habitats have been facing intensified competition due to recovering whale populations, variable prey supply due to environmental perturbations, climate-induced ecosystem changes, and a rise in marine pollution and chronic ocean noise [38, 39, 41, 98–101]. As a remote and largely undeveloped neritic habitat with strong connections to terrigenous nutrient inputs [102], the KFS may have been insulated from many of these trends. If the return to the KFS was prompted in part by its relative quality, then the KFS may be playing a significant role in supporting the recovery of the shelf subpopulation.

#### Recent trends

The stabilization and subsequent decline we observed in the local fin whale population following 2013–2014 (Fig 3A) serves as this narrative’s final, unfinished chapter. The decline in the number of individuals using the area, as evidenced by photo-identification, may seem at odds with the high numbers of detections from shore-based stations in recent seasons (Table 1). However, these patterns could be explained by the intensifying use of the area by a core population of seasonal residents and a concurrent decline in the number of non-resident individuals transiting the area. Our time series is still too brief to draw any conclusions. The trends we have observed may reflect ‘normal’ interannual variation in fin whale distribution, or maybe even a longer-term cycle in which fin whales alternate between prevalence and absence every decade or so. But it may also be indicative of some change in the relative quality of KFS habitat, e.g., better conditions elsewhere in this population’s foraging range, or it could reflect broad-scale disruptions to the distribution and ecology of fin whales in the region. The decline corresponds in time with an unusual series of climatic and oceanographic perturbations, including a 2014 flip in the Pacific Decadal Oscillation from a strong negative phase to a strong positive phase, a strong El Niño–Southern Oscillation event in 2015, a backdrop of perennial sea surface temperature rise, and the establishment of a ridge of high pressure over the northeast Pacific that caused anomalously warm waters to develop in December 2013 and did not dissipate until November 2015 [103–106]. This “warm blob” was documented by Fisheries and Oceans Canada weather buoys stationed within and adjacent to the KFS in 2015 [61], but its impacts elsewhere in the northeast Pacific could also influence the use of the KFS by far-ranging whales. These climate anomalies have been implicated in a wave of mortality in seabirds [107, 108], as well as the 2015–2016 Unusual Mortality Event of fin whales and humpback whales in the Gulf of Alaska and British Columbia, which included stranding events near the entrance to the KFS [109, 110]. Continued research in the KFS and adjacent mainland inlets will position us to monitor the impacts of future perturbations in the years to come.

#### Recovery ecology

The non-exclusive form of seasonal residency exhibited by KFS fin whales, in which individuals rely on the KFS as a regular destination within a larger spatial pattern of habitat use, differs from the strategy of sustained occupancy practiced by the resident humpback whales of this area [111]. This is just one of many site use differences found in these two sympatric populations within the KFS [48–50]. These two populations also differ starkly in size. Humpback whales here are much more numerous [48, 111], which is curious considering that relatively few humpback whales were taken or even recorded in sighting logs during the whaling era (*S1 Appendix*). This discrepancy could simply be due to the whaler’s preferential attention to the larger and more lucrative species, or to the inaccuracy of the logs. It is also possible that humpback whales may have been depleted by prior decades of whaling by the time whalers began using the KFS, or that they were caught further south along their migratory route, before they reached the KFS feeding area. But another possibility is that fin whales were once much more abundant. After all, fin whales were hunted in the greatest numbers, both in British Columbia [47] and worldwide [88], before which they were the world’s most abundant baleen whale [112, 113]. Bayesian models estimate that in the pre-20^th^ century north Pacific, fin whales were four times as numerous as humpback whales [112].

If fin whales were in fact more common in the KFS than humpback whales one hundred years ago, then the comparatively small present-day population of fin whales may indicate that (1) their repopulation of the fjord system is not yet complete; (2) their repopulation was obstructed by the earlier establishment of humpback whales in the fjord system, whose return was documented several years prior to 2005 [114], or (3) their repopulation was obstructed or displaced on a larger scale than that of the KFS. As populations recover within a changing ocean, the spatial and ecological niches they once occupied are unlikely to remain available or unchanged [97, 115]. Despite the centuries of knowledge regarding whale activity in the KFS, it is probable that the whale dynamics we observe in the KFS today are without historical precedent.

### Habitat selection

The fact that the KFS is western Canada’s only fjord system to be used regularly by fin whales, both currently and during the decades of 20^th^ century whaling, begs the question why this is the case. In a study of habitat preferences within the KFS based upon the same line transect data we analyzed here, Keen et al. (2018) posed several hypotheses that might explain the fin whale’s unique use of the KFS: (1) Portions of fin whale habitat in the KFS are remarkably deep. In fact, Squally Channel, the waterway with the highest density of fin whales, is the deepest part of the mainland coast (700+ m, NOAA 2013). It is possible that this particular fjord system is attractive to fin whales for the ways in which it is *unlike* a shallow coastal area. (2) The protected waters, low wave energy, steep fjord walls, and relatively low densities of shipping traffic establish high-quality acoustic habitat in the KFS. (3) The adjacency of the KFS to Moresby Trough, an extensive seafloor depression in Hecate Strait used heavily by fin whales, may deliver nutrient- and larvae-rich waters into the fjord system via tidal pumping.

Adding to these hypotheses, Qualls [116], in her study of euphausiid distributions in the KFS in 2013–2015, found that 99% of the krill community was composed of two species: *Euphausia pacifica*, a species that practices large diel vertical migrations and prefers deeper waters typical of the outer shelf, and *Thysanoessa spinifera*, a species common on the inner shelf and the largest and most lipid-dense krill species in the region [117–119]. These two species were the most common prey found in the stomachs of the whales killed in BC waters [3, 34]. Qualls [116] attributed the co-occurrence of these two species in the KFS to its juxtaposition of unusually deep basins and shallow sills, which satisfy distinct habitat requirements and also facilitate inordinate primary productivity. The sympatry of *E*. *pacifica* and *T*. *spinifera* in the KFS may be providing fin whales with a larger, more reliable, and more seasonally extensive food supply compared to the continental shelf and other mainland inlets. We suggest that this uncommon euphausiid assemblage adds to the attraction of the KFS as a foraging ground.

The confined distribution of fin whales within the KFS also remains to be explained. Fin whales use much less of the fjord system than do humpback whales [50, 64]. This may be a matter of preference or interspecific habitat partitioning [50], but it may be due to delayed range expansion [50]. During the whaling years, fin whales were seen and caught further into the fjord system than they typically venture now (S4 Fig in S1 File; Ernie Hill, pers. comm. to JP), though this may have been a function of effort or the result of driving fin whales further into the fjord system during chase. It is possible that their habitat preferences may still be in a state of flux (see ‘Recovery Ecology’ subsection above). Euphausiid distribution may also be informing fin whale distribution within the KFS; the highest *T*. *spinifera* densities observed by Qualls [116] overlap with the primary fin whale habitat we identified here (Fig 5).

It is noteworthy that the two areas of highest fin whale density, Caamaño Sound and Squally Channel (Fig 1A), are starkly different habitats. The former is a broad and relatively shallow (< 200 m) basin exposed to strong winds and waves from Hecate Strait. The latter is a relatively narrow, deep (> 600 m) passage that is calm and free of swell throughout most of the summer. Affinity for these two areas cannot be driven by the same associative patterns with habitat features, which leaves us with three viable hypotheses: (1) fin whales are associating directly with prey distribution, without reliance upon physiographic proxies; (2) fin whales practice two separate strategies of habitat use within the KFS, possibly driven by prey switching between *E*. *pacifica* and *T*. *spinifera*; and (3) habitat preferences in the KFS may be driven not by physiographic, oceanographic, or ecological associations, but instead by memory-driven site fidelity to specific areas. This third option is supported by evidence of repeated departures and returns, wherein whales could be repeatedly checking on the productivity (or some other quality) of known areas.

### Management considerations

The seasonal and spatial whale distributions we observed are relevant to marine traffic management in the KFS. The Inside Passage traffic route, which transits the Kitimat Fjord System to the north of Gil Island (Fig 1A), is outside of present-day primary fin whale habitat (Fig 5). However, an alternative route, known to some as the ‘Outer Inside Passage’, is transited by commercial and recreational vessels to the northwest-southeast through Caamaño Sound (Fig 1A), one of the most heavily used areas for fin whales (Fig 5). Additional shipping routes have been proposed for Caamaño Sound as well as Squally Channel, which is the waterway with the greatest number of fin whale detections (Fig 1B–1E), the highest encounter rates (Fig 5), and the greatest spatial density (S6 Table in S1 File). If all proposed shipping projects currently engaged in environmental review are permitted to proceed, large vessel traffic through this fin whale habitat will increase in 2024 by 13-fold [45, 46, 120]. The confined waterways of this fjord system concentrate traffic and reduce ship maneuverability, which may lead to higher rates of ship strike compared to the open ocean. Given that fin whales are the most commonly struck baleen whale in the northeast Pacific [31, 121] and already face unsustainably high strike mortality rates along the west coast of North America [31], heightened management measures may be necessary to protect the fin whales using this unique habitat. Preliminary collision risk assessments have been prepared for this area [122], but updated analyses that incorporate findings presented here, such as seasonal abundance patterns and spatial density estimates, are urgently needed.

Federal mechanisms for enforcing vessel management within this fjordic fin whale habitat have been complicated by the recent proposed downlisting of the Canadian Pacific fin whale stock from Threatened to Special Concern [3]. Whereas Threatened status specifically prohibits the destruction of critical habitat and affords other protections, Special Concern requires only the drafting of a management plan [123]. Downlisting was recommended based upon large numbers of fin whales that have recently been observed in offshore waters [3], but this change is likely to affect the shelf subpopulation of about 400 individuals, of which the KFS fin whales are a part [36], quite disproportionally. This subpopulation exhibits limited interchange with offshore habitats and strong site fidelity to the coastal zone, where the region’s highest densities of pollution, vessel traffic, and ocean noise occur [3, 36, 43].

Fin whales’ use of this fjord system, in particular their itinerant seasonal residency, underscores the need to frame concepts of habitat importance with care. It is possible to construe such residency patterns as an indication that the habitats they use are unimportant or easily replaced. However, places need not be occupied on a constant basis in order to be deemed critically important. Consider the importance of nesting trees for passerine birds, flowing rivers for anadromous fishes, or grocery stores for urban humans. For massive pelagic predators like the fin whale, population survival hinges upon access to a network of reliably productive foraging areas. And, of equal importance, population resilience depends upon (1) redundancy in the habitats upon which they rely, (2) diversity within those habitats, and (3) a plurality of habitat use strategies throughout the population [124, 125]. Furthermore, fin whales of the KFS are known to adhere to feeding thresholds in which only unusually productive conditions can sustain their energetic needs [48]. For threshold foragers, finding satisfactory prey patches requires extensive searching in which only a small portion of the habitat *accessible* to the predator will actually prove *useful*. It follows that the areas of demonstrated long-term value to these fin whales are unlikely to be readily replaced.

The fin whales we have observed in this fjord system represent a rare and perhaps even unique dimension of this species’ natural history. Species are often characterized by general rules and majority patterns, but in actuality each is highly dimensional and richly diverse [126]. But species- and stock-oriented management can, in an effort to save the whole, fail to preserve the sum of its parts [126–128]. An ecosystem-based approach to conservation, which emphasizes the value of unique ecological scenarios, may prove more effective at indirectly protecting the richness of each single species [128–130]. The marginal and peculiar exceptions, such as the fin whales of the Great Bear Rainforest, contribute to more than the resilience and versatility of a recovering species; without them, a species cannot, in any complete sense, be saved.

## Concluding remarks

Beyond continued monitoring of the population size, distribution, and seasonal presence of KFS fin whales, future research will allow us to investigate further into several inconclusive patterns apparent in our results: the gregarious but fluid social network of these fin whales, which to our knowledge is the first study of sociality in *B*. *p*. *velifera* but is similar to that observed in *B*. *p*. *physalus* in the Gulf of St. Lawrence [131]; evidence of preference for the Caamaño Sound area by mother-calf pairs (S13 Fig in S1 File); evidence consistent with either a seasonal or interannual shift in habitat use from exterior to interior waterways (S11 Fig in S1 File); the similarity in body size between present-day fin whales using the KFS and those reported during whaling (Fig 8); and the apparent contrast between the fin-biased catch record and the humpback-dominant reoccupation of the fjord system. Other than these priorities, the recent establishment in Squally Channel of a seafloor hydrophone array capable of localizing and tracking calling fin whales presents a unique opportunity to study these fjordic fin whales and their interactions with vessels using concurrent acoustic and visual methods [52, 132, 133].

This study is a product of the long-term collaboration of indigenous, federal, non-profit, and academic research teams, as well as the generosity of database managers from public and private sectors. All of these methodologies and data sources were required to reconstruct and interpret this narrative. We believe this study demonstrates the value of place-based, collaborative science in regional conservation. As recovering populations reckon with increasingly altered marine habitats, we anticipate that networks of local research partnerships will play an essential role in ecosystem monitoring, threat assessments, and management efforts.

## Supporting information

S1 Appendix Whales and whaling in Caamaño sound. (PDF)

S1 File This supplementary file contains additional tables and figures referenced in the main text. (DOCX)

S1 Data (CSV)

S2 Data (CSV)
