# Correction: Fin whales of the Great Bear Rainforest: *Balaenoptera physalus velifera* in a Canadian Pacific fjord system

**DOI:** 10.1371/journal.pone.0305297

**Published:** 2024-06-21

**Authors:** Eric M. Keen, James Pilkington, Éadin O’Mahony, Kim-Ly Thompson, Benjamin Hendricks, Nicole Robinson, Archie Dundas, Linda Nichol, Hussein M. Alidina, Hermann Meuter, Chris R. Picard, Janie Wray

There are errors in S1 Data. It should have been caption as Data obtained from the B.C. Cetacean Sightings Network were collected opportunistically with limited knowledge of the temporal or spatial distribution of observer effort. As a result, absence of sightings at any location does not demonstrate absence of cetaceans or sea turtles.

Moreover, the records from one of the data contributors should have been labelled as ‘Opportunistic’. Please view the correct S1 Data below.

## Supporting information

S1 Data (CSV)
