# Supplementary material for: Fin whales of the Great Bear Rainforest: Balaenoptera physalus velifera in a Canadian Pacific fjord system
Source: PLoS One. 2021 Sep 3;16(9):e0256815. doi: 10.1371/journal.pone.0256815 (PMC8415578; doi:10.1371/journal.pone.0256815)
Supplement: S1 Appendix — (PDF) [file pone.0256815.s001.pdf]

Supplementary Appendix

to Keen, Pilkington, et al., “Fin whales of the Great Bear Rainforest: *Balaenoptera physalus velifera* in a Canadian Pacific fjord system.” Journal (2021)

Whales and Whaling in Caamaño Sound

An historical perspective

Authors’ note:

This appendix was written primarily by author James Pilkington as part of focused research on the history of Caamaño Sound, a major waterway in the outer channels of the Kitimat Fjord System (KFS). Caamaño Sound is where most of the area’s commercial whaling effort took place.

While the focus of this appendix is Caamaño Sound, the research involved reading accounts of whalers from deeper within the KFS, and the whaling records summarized here also include kills that occurred outside of the KFS proper, particularly near the entrance to Caamaño Sound in southeast Hecate Strait, as well as records for species other than fin whales.

Hence, while the historical account of whaling detailed here will apply to the relevant sections of the main text, the catch totals and demographic composition of catches reported within this appendix differ slightly than those reported in the main text.

Please refer to Figure 1 in the main text for the locations of place names mentioned in this appendix.

Whales and Whaling in Caamaño Sound

An historical perspective

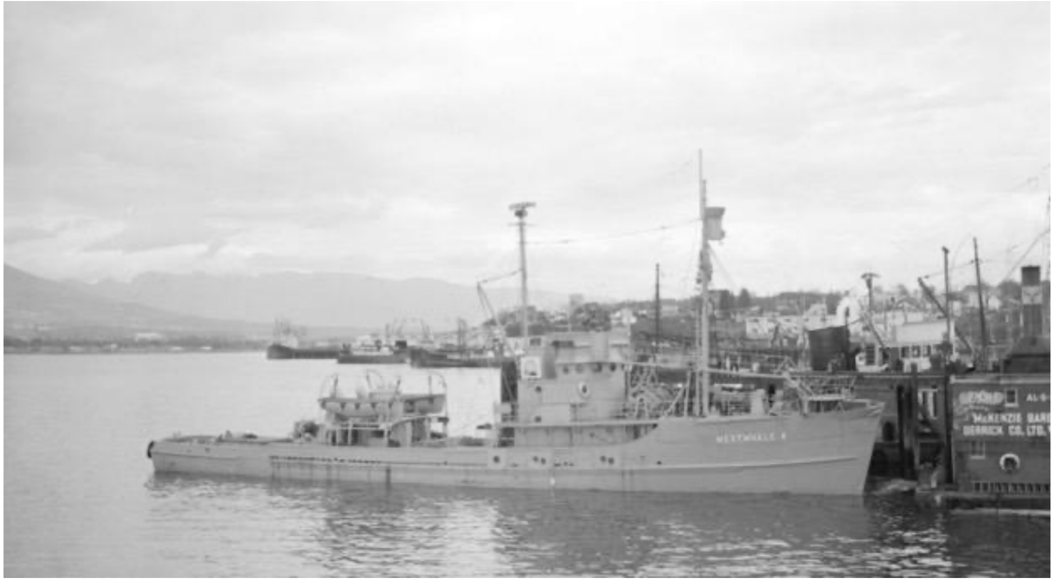

Figure 1. *Coal Harbour catcher boat, WestWhale 4, wintering in Vancouver harbour, 1960s. This vessel killed more whales in the Caamaño Sound area than any other catcher boat that operated there. City of Vancouver Archives, Ref AM1506-S3-S-: CVA 447-8935)*

Introduction

Recent research efforts in the Caamano Sound region have highlighted the importance of this area to several populations of whale (Fin whales: Keen and Pilkington et al. 2021 (this manuscript), Ford and Nichol 2018; Humpback whale: DFO 2013, Ashe et al. 2013, Keen et al. 2017, Wray & Keen 2020; Resident Killer Whale: Ford 2006, Pilkington et al 2011; Bigg's Killer Whale: Ford et al. 2013). Although today's research makes a strong argument for Caamaño Sound as an important area for a variety of whale species, historical information on whale presence in this area has not been collectively summarized but would provide valuable insight and context for the present-day occurrence of whales in the area. In light of this, I (JP) undertook a review of historical documentation relevant to the area that may allude to the prior presence of whales in the region, including: log books from early explorers and fur traders who visited the region, historic commercial whaling records from this area, and cultural impact statements summarizing some of the cultural practices and traditional knowledge of First Nations of the region. Of interest were early explorers' observations of whales, local First Nation's traditional use and observations of whales, the species and quantities of whales taken and sighted by whalers, the number of whaling ships that worked there, the biological data collected from the individuals killed, and any anecdotal information describing how whaling was undertaken in the Caamaño Sound area.

Methods

Early Accounts and Place Name Origins

I reviewed several cultural impact statements (namely Marsden 2012, Satterfield 2012, and Menzies 2011), that were written on behalf of the the Gitga'at and Gitxaala First Nations as official evidence examining the potential cultural impacts of proposed shipping expansion projects that would substantially increase large ship traffic through their respective territories (their traditional territories are the primary ones that overlap with our study area). These official statements focus on describing the complex cultural practices of these Nations, however, the cultural practices of these two Nations are inherently and utterly entwined with the natural history of their respective territories, and thus, the reports represent a way to examine pre-contact natural history of the region through a cultural lens.

Co-author, Janie Wray also asked the late Gitga'at Elder, Ernie Hill, whether the Gitga'at people used to hunt whales.

To assess cetacean presence prior to the onset of whaling activities, I undertook an examination of the journals kept by the most prominent explorers and fur traders known to have visited the Caamaño Sound area during the late 18th and early 19th centuries, namely Captain James Colnett (1787), Captain Jacinto Caamaño (1792), Archibald Menzies (1793), Captain Joseph Bishop (1795), and Captain Daniel Pender (1866-68) (years in brackets represent the year(s) when the study area was visited) . Note, it was not practical to access the journal of Captain Joseph Ingraham (1789) within the timeline for this report, even though he traded in the study region. None of these parties had a mandate to reliably document whale sightings, but any anecdotal observations of cetacean activity within the Caamaño Sound area found in these journals are summarized in this report. Indications of local natural history in charted place names (both, currently used, and early charted names that were not officially adopted) was also noted.

Whaling information

To summarize the documented catches of whales taken during the early "Yankee" whaling period in the North Pacific off British Columbia during the 1800's, we used the American Offshore Whaling Logbook Data (American Offshore Whaling Logbook Data, https://whalinghistory.org, Mystic Seaport Museum, Inc. and New Bedford Whaling Museum – most recently accessed March 24, 2021), as well as examining relevant literature on the topic of early American whaling in the North Pacific. Of importance were kill or sighting locations in the vicinity of the Caamaño Sound area.

To summarize the documented catches of whales taken by shore station efforts throughout the 1900's off British Columbia, we used data from the *B.C. Historical Whaling Database* (HWD) maintained by Linda Nichol of the Cetacean Research Program at the Pacific Biological Station, Nanaimo, BC (Nichol et al. 2002). The database was queried for all catches of all species that occurred within a geographical boundary that encompassed Caamaño Sound, including northward extending waterways up to Douglas Channel, as well as the seaward approaches to the entrance of Caamaño Sound from Hecate Strait. The HWD encompasses information collected on nearly all documented cetacean catches and sightings made during all shore-based and pelagic whaling operations that occurred in British Columbia, from 1908-1967. Data from pelagic catches carried out by the former Soviet Union in the 1990s were omitted due to falsification of the data (for more information, *see* Nichol et al. 2002).

The following information about whales killed by whalers in Caamaño Sound was sought from the HWD: location of kills, name of catcher ships that made each kill and the vessels' originating shore station, the whale's species, overall body length, sex, fetus presence/absence, fetus sex and length, description of stomach contents, and the number of individuals in the group in which the killed whale was sighted. Sightings of whales were also documented by whalers, and these sightings were also used in this analysis, including the following information: species, group size, and location.

In addition to this data collection, we calculated a crude estimate of the effort conducted by whaling ships in Caamaño Sound. The actual effort of whaling boats in the area was impossible to quantify because ship locations were only updated in the log books once a kill was made; there is no measure of how much time the ships spent searching in an area prior to making a kill. Navigation logs in which the captain updated positions regularly and made notes of ship activities are extremely rare, many have been lost or destroyed. However, one navigation log for the *West Whale 4* held in the HWD support material was examined for this report and provided invaluable—albeit limited—insight into how whalers used Caamaño Sound. Because these ships were fast and could travel great distances in a single day (Caamaño to Coal Harbour, Vancouver Island, in less than 24 hours), we could not simply assume that days between kill days were still spent in Caamaño Sound. However, because the ships noted their locations with every kill and/or sighting of a whale, we were able to glean a fairly crude measure of the minimum effort conducted there, measured in *boat days*. We defined a boat day as a day in which one or more sightings and/or kills were made within the boundaries of our focal area, but cumulative by the number of boats (eg. two vessels reporting kills on the same day would equal 2 boat days). By comparing the boat days to the number of kills made, we calculated crude catch per unit effort values (CPUEs).

Early accounts and place name origins

The earliest European documentation of whales in the area of Caamaño Sound came from Captain James Colnett of the fur trading ship *Prince of Wales*. In September 1787, after crossing to the mainland coast from Haida Gwaii to find new fur trading opportunities (otter pelts), Colnett made note in his journal of a "[q]uantity of Whales blowing in all Quarters" (Dubois 2005, p. 139) as his vessel came out of persistent fog while approaching the southern portion of Banks Island approximately two miles off today's Cape Terror (Southwest tip of Banks Island).

Five years later in 1792, Don Jacinto Caamaño was tasked by the Spanish government to explore the mainland coast north of Vancouver Island and search the areas around 53°N (in the vicinity of current day Caamaño Sound) for the mythical passage of Admiral De Fonte, a passage rumored to extend west to east through North America ending in the Hudson's Bay[1]. Aboard the Spanish ship *Aranzazu*, anchored off of a First Nation village (Citeyats) at the Cherry Islets (Northern Squally Channel), Caamaño and his men were preparing the boats to survey the inlets at daylight when:

> "The [C]hief of the village, noticing these preparations, came to ask me [Caamaño] whither the boats would be going. On my telling him that they were to explore and survey the various channels, he explained to me by signs that these were innumerable, ran inland a great distance, and were infested by huge animals that

[1] Captain Charles Duncan, sailing the *Princess Royal* with Colnett's *Prince of Wales*, alluded to the possible presence of the fabled passage in Douglas Channel in 1787 during a meeting between the Spanish and British in Nootka Sound (Wagner & Newcombe 1938).

> thrust the whole body out of water, attacked and over-set the natives' canoes and devoured their occupants." (Caamaño's Journals, p. 275)

Caamaño did not believe these stories[2], but it is entirely conceivable that the animals referred to were Humpback whales, which are known to concentrate in large numbers in this fjord system. Particularly in the fall, the whales engage in competitive displays prior to migration involving very active surface behaviours such as breaching and lob-tailing. Interestingly, Jacinto Caamaño was there throughout October, prime season for Humpbacks to be in this area engaging in such behaviours, which could be what the Chief was describing. Also, today, there are a few 'friendly' Humpbacks in this region that are known to approach vessels and even engage in rubbing on the boats or conduct aerial displays in close proximity. Certainly, native canoes, and their occupants, would not have faired well if a whale breached or lobtailed on or near them, which undoubtedly could have fueled such stories of people being 'devoured'. It is also possible that Caamaño misinterpreted the Chief's gestures, which could have instead described the action of groups of Humpbacks bubble net feeding, which is common in the area today (including northern Squally Channel where Citeyats was situated). Today, a dozen or more humpback whales engage in bubble net feeding in one or more large groups during which they break through the surface together with their mouths agape , occasionally in close proximity to unsuspecting vessels, just as the Chief's reported actions depict.

In 1793, not one year after Caamaño's explorations, captain George Vancouver conducted explorations of the Caamaño Sound area during his 1791-1795 expedition. Archibald Menzies, the surgeon and naturalist aboard Captain George Vancouver's vessel, *Discovery*, kept notes on natural history observations during the voyage, On July 3, 1793, he wrote: "several whales and porpoises were seen passing through the channel with the tide" (Menzies 1793). The vessel was anchored just east of Whale Channel in McKay Reach at the time of this observation, between Princess Royal and Gribbell Islands.

Place names can also be an indication of significant aspects of an area's natural history. Whale Channel, which separates Gil Island from Princess Royal Island, was officially named in 1870 by 2nd Master Daniel Pender during his survey of the area (1867-1870) aboard the *S.S. Beaver,* chartered by the British Admiralty. The reason for the naming is unknown, and there is no mention of it in his log book. Prior to Pender, it appears that Jacinto Caamaño had also named the same channel. The ink on Caamano's original canvas chart has bled badly, but the handwriting for this channel's name seems to read "Paso Ballena" (unable to confirm). If so, Pender likely honoured (albeit anglicized) this earlier naming of Whale Channel. The name is assumed to be a result of observations of, experiences with, or stories from First Nations of whales during their times in the area (such as the story told to Caamaño by the Chief at Citeyats in Squally Channel).

Fin Island, in north Squally Channel, is also an obvious reference to whale activity in the area. No information on the origins of this name could be found, though it is likely in reference to the shape of the island, which looks like a dorsal fin when viewed from the north.

All of these observations and anecdotes allude to the presence of whales in the Caamaño Sound area well over a century prior to the onset of commercial whaling there, and nearly two and a half centuries prior to present.

---

[2] "I had no doubt of the untruth of this story, in spite of the chief's expressive gestures, and confirmation by all the other Indians." (Caamaño's Journals, in Wagner and Newcombe 1938, p. 276)

First Nation interactions with Caamaño Sound whales

The traditional territories of Gitga'at First Nation and, to a lesser extent, Gitxaala First Nation, encompass the waters and lands of Caamaño Sound and surrounding fjords. According to the Gitga'at, the claiming, use, stewardship, and defence of these territories has occurred since time immemorial. It is plainly evident that GItga'at and Gitxaala cultures are wholly entwined in and inseparable from their relationship with the natural history of these territories (Marsden 2012, Menzies 2011). An examination of literature describing various aspects of Gitga'at culture, from orthography and traditional place names to food use and phenology, highlights that the area's indigenous people had a knowledge of and relationship with whales in this area well before the arrival of Europeans (potentially dating back millennia). The following points substantiate this, but in no way represent an exhaustive examination of the relationship of Gitga'at or Gitxaala peoples to whales:

- Although several First Nation groups along the west coast of North America are known to have actively hunted whales for subsistence (Beland, 2014; Monks et al. 2001, Losey et al. 2007, Ford 2014), the late Gitga'at elder Ernie Hill has noted that the Gitga'at people did not hunt whales, simply because of the sheer abundance of other wild foods available to them in their territories. However, it was noted that whenever the Gitga'at people found a dead whale, they would take advantage of the scavenging opportunity and consumed and/or used the animal.[3] In comparison, Menzies (2011) notes that the Gitxaala harvested whales for food and oil prior to and at the time of European arrival, but no information was in the report regarding how frequently and where they hunted.

- In 1792 while storm-bound off the large village of Citeyats (Cherry Islets, Squally Channel), Jacinto Caamaño was invited ashore as a guest of honour of the most prominent leader of the Gitxaala people – Smoygyat Homstits. In his journal, Caamaño noted the use of "whale bone" in the ceremonial clothing of the Chief during a ceremony he witnessed while ashore[4]. However, it is uncertain whether the specific whale materials that the Chief was wearing were acquired through trade with other groups, scavenging, or hunting, and from what species the materials originated. Nonetheless, the integration of whale materials into the ceremonial clothing of local indigenous peoples highlights a relationship with whales that predates the arrival of Europeans. In fact, archaeological studies undertaken by the Gitxaala at Citeyats show that the village was continuously inhabited at least 4,000 years before present (Menzies 2011).

- Whales are culturally important species to the Gitga'at people as highlighted in cultural impact studies. Satterfield et al. (2012) note that Fin, Humpback, and Killer whales are all rated with maximum scores in 'Knowledge use' and 'Knowledge exclusivity' within Gitga'at culture. Fin, Humpback and Killer whales are also listed as "Critically Important" to Gitga'at knowledge transmission by being represented strongly in oral history/*adawx* (the oral history passed on through generations delineating the cultural practices and history of the Gitga'at people) (Satterfield et al. 2012).

- The Gitga'at Nation's traditional place name for McKay Reach, Lutguk'laxne'ex, roughly translates to "Where Around on Killer Whales (Fins?)" (Marsden 2012), highlighting the long-

[3] Personal communication, Ernie Hill to Janie Wray.

[4] *"On his head a large well-imitated representation of a seagull's head, made of wood and coloured blue and pink, with eyes fashioned out of polished tin; while from behind his back stuck out a wooden frame covered in blue cloth, and decked out with quantities of eagles' feathers and bits of whale bone, to complete the representation of the bird." (*Caamaño's Journals, p.291).

term presence of this cetacean species still common in the area today (Ford 2006, Ford et al. 2013, Pilkington et al. 2011).

- Sm'algyax, the predominant language spoken by the Gitga'at and Gitxaala includes words for types of cetaceans (*łbuun* = whale, *'naaxł* = killer whale or blackfish, *dziiw̃* = dolphin and porpoise) (Satterfield et al, 2012).

- One of the three Gitga'at clans is the Killer whale (Marsden 2012).

- One Gitga'at elder noted observations of local whale movements as indicators/predictors for greater regional weather conditions, and would use this knowledge to help inform decisions about travelling safely (Satterfield et al. 2012, pg. 84).

- A Gitga'at name for a location in southcentral Wright Sound, *Xbunaxnox*, translates roughly to "Where Spouts a Naxnox" (Madsen 2012). 'Naxnox' is the Sm'algyax word for 'supernatural being or wonder'. In this case, the translation "Spouts" implies the Naxnox may be of whale origin; however, more information is needed for proper interpretation.

Industrial Whaling

Industrial whaling began in the North Pacific in the early 1830's with the arrival of the 'Yankee' whaling fleets. These early efforts were predominantly of American origin, though dozens of other countries also participated (Webb 1988). These whalers used sail-powered whaling schooners based out of the Hawaiian Islands or California for hunting Right, Sperm, Bowhead, Humpback, and Grey whales over the entire North Pacific, including the waters off current-day British Columbia.

Data compiled from whaling logbooks from this era indicate that they worked all over the outer waters of British Columbia with occasional use of the shelf waters, including Queen Charlotte Sound near Caamaño Sound (Townsend 1935; Maury 1887; Smith et al. 2012; American Offshore Whaling Logbook Data 2021). It is unknown whether these early whalers ventured into Caamaño Sound, but it is evident that they worked in the vicinity, killing at least one North Pacific Right whale off Price Island in July, roughly 50 nautical miles from Caamaño Sound (Figure 3).

Between the mid-1800s and early 1900s, the decimation of easy-to-catch whale species and the invention of the steam engine created the onset of a new era in whaling that allowed whalers to hunt the larger, faster, and as-yet-unhunted species, such as Blue, Fin, and Sei whales (Gregr et al 2000). Generally, catch processing was moved from ships to shore stations that were built in select places along the west coast of North America. Powerful steam-driven catcher boats with harpoon cannons and exploding harpoons (like the one in figure 8, found on a beach on the west side of Campania Island, Caamaño Sound) were used to catch the whales whose carcasses were brought back to shore stations to be processed.

Beginning in 1904, shore stations were being built in several locations along the BC coast, primarily Haida Gwaii and the west and east coasts of Vancouver Island, which sparked competition for the remainder of the unclaimed, yet still lucrative, whaling grounds in northern BC waters. During this time there were three competing proposals to build shore stations in and around Caamaño Sound. The first proposal, which was subsequently revoked, was put forth by Cereno Jones Kelley of Victoria, BC, who requested the whaling rights for several areas of the coast, but included Nepean Sound and Estevan Sound, which are connecting channels to Caamaño Sound (Webb 1988). Another station was proposed for "a location 3 nautical miles north of Gale Point, Banks Island,"

(likely Kooryet Bay in nearby Principe Channel) by the director of the Pacific Whaling Company, Sprott Balcom (who also owned the Rose Harbour, Naden Harbour, and Kyuquot whaling stations)(Webb 1988). The third proposed bid for a station was held by a captain of the Canadian Pacific Railroad, John O. Townsend, and it was to be built on Campania Island in Caamaño Sound, at an undisclosed location. Reportedly, Townsend's plans were ultimately rejected by the federal government because of rumors festered by Sprott Balcom, claiming that Townsend was to "sell out [...] to a Norwegian concern" if given the Campania Island permit (Webb 1988). In the end, Sprott Balcom was granted the permit to build the Banks Island station, but the plan never came to fruition. Incidentally, Balcom's other permitted plan for a station in Fitz Hugh Sound, which would have also allowed catcher boats access to Caamaño Sound, also never became reality (Webb 1988).

Despite these failures to build stations in or nearby Caamaño Sound, the Sound did not escape the pervasive whaling efforts of BC shore stations in the decades to come. Of the five main shore stations that operated in BC, just two were responsible for the whaling that took place in the Caamaño Sound area: Rose Harbour and Coal Harbour. Located near the southern tip of Haida Gwaii on Kunghit Island, 90 nautical miles west across Hecate Strait from Caamaño Sound, the Rose Harbour Whaling Station operated between 1910 and 1943 with various breaks and closures (Nichol et al. 2002). This station only documented taking whales from the Caamaño Sound area in 1926 and 1927, though this station only documented the locations of kills between 1924-1928, which leaves the possibility that more whales could have been taken from the Caamaño Sound area prior to 1924 but the locations were not recorded. The Coal Harbour Whaling Station was located in Coal Harbour, inside Quatsino Sound on northwestern Vancouver Island, and operated from 1948 to 1967 with a break in activity in 1960 and 1961. The Coal Harbour station was the only station in BC to operate after World War II. Faster, more powerful catcher boats than those that operated out of Rose Harbour, with the additional aid from spotter planes meant that nearly the entire coast of BC was in range of this station, and Caamaño Sound - roughly 190 nautical miles away - was indeed a focus of some of their efforts; the vast majority of whaling activity that occurred in Caamaño Sound was accomplished by vessels from the Coal Harbour whaling station.

Whaling Effort

Whaling along the BC coast was very seasonal, typically beginning in April and ending in October or November (Gregr et al 2000). In Caamaño Sound, whaling occurred from May to September, with no indication that any effort took place outside of this period there.

Entries in ships' catch logs were only created when a kill or sighting was made, and did not include notes on navigation, route planning, or days spent in certain areas searching with no success. As a result, the boat days we present here, based off of the catch logs, are only a minimum indication of effort in the area. For a more accurate measure of effort in areas, 'ships logs' or 'navigation logs' would be required. Such logs noted periodic location updates, weather, sea state, and ship activities, however, many of these logs have been lost.

A total of 87 boat days were conducted in Caamaño Sound between 1926 and 1967 (see Tables 5 & 6). A crude catch per unit effort (CPUE) in *kills per boat day* was calculated for each year and ranged from 1 to 2 (mean=1.4). Seasonally, boat days occurred from May through September, but were concentrated towards the later part of the season, namely August and September. It is uncertain whether this is solely because more whales were present in August and September resulting in more kills entered in the log, or whether boats visited the area more readily during that time of year resulting in more kills then. In total, 13 different whaling ships made at least one kill in

the Caamaño Sound area between 1926 and 1967, but other ships may have hunted there unsuccessfully, their effort going undocumented. The number of different ships to have made a kill in Caamaño in any given season ranged from 1 to 4 (mean=2.8) (see Table 2).

Notes found in catch logs and one navigation log indicate that whalers had a knowledge of Caamaño Sound's abundance of whales and routinely made efforts to travel directly to Caamaño Sound for the purpose of exploiting it. The 1964 navigation log of the *West Whale 4*, a vessel that routinely hunted in Caamaño Sound, has information not included in the database, which illustrates this. At 1450 on September 21 1964, the vessel left Kain's Island at the mouth of Quatsino Sound (West Coast of Vancouver Island) and travelled a direct course for Caamaño Sound. The vessel arrived at McDonald Island just outside Caamaño Sound by 0900 on September 22, approximately 16 hours later (a distance of 190 nm). They entered Caamaño Sound in rain and fog, made note that they saw no whales[5], and made their way to Barnard Harbour (Whale Channel) for the night where they tied up to *West Whale 8*, already anchored there -- a vessel that had killed three Fin whales from a group of ten in Caamaño Sound just two days before. On the 23rd, the *West Whale 4* made its way back into Caamaño Sound from Barnard Harbour in rain and fog, chased a Fin whale unsuccessfully in Laredo Channel, then continued south to McInnes Island[6].

These notes highlight several interesting points. Whaling vessels travelled to Caamaño Sound intentionally, but at least in this case, were quick to leave if few whales were sighted. Whaling vessels used Caamaño Sound without successfully making kills or sightings, thus undertaking effort that is not included in the database -- in this case, at least two boat days. Additionally, the notes say *West Whale 4* chased a Fin whale unsuccessfully on the 22nd as it left the Sound, but this was not logged properly as a sighting in the sightings log, having only mentioned the event in the ship's navigation log. As such, there is no record of the event in the database. Also, *West Whale 8* made three documented kills in Caamaño on September 19th, equating to one boat day of effort, but was still in Caamaño on the 22nd when the *West Whale 4* arrived (having not made any kills or sightings between the 19th and 22nd). Had *West Whale 8* conducted effort in Caamaño between the 19th and 22nd, or had they gone somewhere else and come back? These uncertainties are only some of the challenges faced when trying to quantify historical whaling effort.

Numbers and Species of Whales Taken

20th Century Commercial Shore-based Whaling in BC
The intensive efforts of shore-based whaling in British Columbia resulted in a minimum of 24,427 whales caught all over the BC coast (Nichol et al. 2002). A total of 129 kills were made in the Caamaño Sound area, representing 0.5% of the total documented BC catch. Kills in the Caamaño Sound area were comprised of five different species: Fin, Humpback, Sperm, Sei, and Blue whale (see Table 1). Fin whales represented the vast majority of the catch, encompassing 75% (n=97) of the whales killed, followed by twenty-five Humpback whales, four Sperm whales, two Sei whales[7] and one Blue whale.

Fin whales were taken throughout the Caamaño Sound area, with concentrations occurring in inner Caamaño Sound and around Gil Island, as well as a diffuse distribution in the western open

---

[5] *Entry for 11:00 September 22, 1964 "Caamaño Sound - no whales seen. Rain [and] fog"*

[6] "11:00 - Chase 1 fin - Laredo Channel"; "16:00 - Lost fin - Head for outside"; "17:30 - McInnes Island"

[7] *During the Rose Harbour era, identification of Sei whales may have been questionable due to their similarities to Fin whales and the general lack of effort for Sei whales during this earlier era in BC (Gregr et al. 2000; a market for Sei whale products began during the Coal Harbour era). One of two Sei whales caught in Caamaño Sound was caught during the Rose Harbour period and so may be viewed with less confidence.*

portions of the Sound adjacent Hecate Strait (Figure 3). Humpback whales were primarily taken from Campania Sound and lower Whale Channel around Gil Island, with a few taken in outer Caamaño Sound. One of the two Sei whale kills occurred well offshore of Caamaño Sound at the most southwestern extent of our focal area, whereas the other kill occurred just a few miles west of Rennison Island. The only Blue whale caught in this area was taken well offshore of Caamaño Sound at the westernmost extent of our focal area adjacent Hecate Strait on the edge of Moresby Trough. Three of the four Sperm whales caught in the Caamaño Sound area were well inside the inner coastal waterways of the Sound, one in Campania Sound between Campania and Princess Royal Islands, and two in the northern portion of Squally Channel near Otter Channel. The fourth Sperm whale was caught in the western extremities of our focal area.

The largest number of whales to be killed in a single day in this area was five Fin whales, which occurred twice, once in July 1955 and again in August 1963.

Whaler Sighting Records

Beginning in 1963, whalers logged visual sightings of whales in addition to kills made. In combination with kill data, these sightings help provide a better indication of the actual numbers of whales present in some areas, however, sightings were not consistently documented, and no effort data is associated with them. Nonetheless, whalers documented 273 whales in 56 sightings in the Caamaño Sound area between 1963 and 1967 (Table 2). In contrast, just 73 whales were killed over the same time period in the same area. Fin whales were the most abundantly sighted species, accounting for 94% of total individuals sighted. Fin whale group sizes ranged from one to 15, with an average of five. Eleven Humpback, two Sei, and four Sperm whales were also sighted. It is uncertain whether whalers were selective in recording sightings for particular species, possibly favouring to document sightings of more desirable species (e.g. Fin whale over Humpback whale).

Seasonality of Catches and Sightings

There is currently no way of telling how strongly the seasonality of catches and sightings in Caamaño Sound is biased by effort. Considering this, a summary of catches and sightings of each species by month is presented in Tables 3 and 4, respectively.

The majority of catches took place in August (49.6%, n=64) with 79.7 per cent (n=51) of August kills being Fin whales. Humpback whale catches also peaked during August, the peak extending into September, with 10 and 11 kills, respectively.

Sightings were documented for each month of the whaling season except April with peak numbers of individuals sighted in August (n=135) and September (n=80). Whales sighted in August encompassed 49.4 per cent of monthly whales sighted, with the vast majority of August sightings being Fin whales (91%). Similarly, the vast majority of whales sighted in September were also Fin whales (96%).

Life History Data Collected on Catches

Length and Sex of Catches

Both male and female Fin and Humpback whales were caught in Caamaño Sound. All Sperm and Sei whales caught were males, and the only Blue whale caught was female. Mean lengths of both sexes of Fin and Humpback whales from Caamano Sound are consistent with the mean lengths of the total BC catch of each species, respectively, for both sexes reported by Gregr et al. (2000) and

suggest that both immature and physically mature animals were caught, and three of these Fin whales at a length of 68 ft were amongst the largest taken in British Columbia. The lengths of all Sperm and Sei whales taken were typical of mature males of these species (Gregr et al. 2000). The length of the only Blue whale taken suggests the animal was immature, which also aligns with lengths of the total BC blue whale catch (Gregr et al. 2000)

Fetus Presence and Length

Fetuses were present in four female Fin whales and one female Humpback whale. The documented Fin whale fetus lengths ranged from 72 to 153 inches, while the only Humpback whale fetus measured 57 inches. All Fin whale fetuses were found in whales larger than 60 feet long, while the Humpback fetus was from an individual of 39 feet.

These fetus lengths of both Humpback and Fin whales are smaller than reported length-at-term for the species (Gregr et al 2000), suggesting that birthing was not taking place during the summer time around Caamaño Sound.

Mammary Condition

Table 9 presents the condition of mammary glands of 23 female whales killed in the study area: 21 Fin and 2 Humpback whales. Evidence of lactation (traces of milk) was found in two of the Fin whales caught, both were killed in early August. This suggests that these whales were either nursing calves at time of death or had weaned calves relatively recently prior to death. The majority of Fin whales, and two Humpbacks, caught were “nonparous”, which suggests they were: immature, pregnant for the first time, or had ovulated but had not yet become pregnant. Lengths of the nonparous individuals suggest that there were representatives from each of the previously mentioned scenarios (FWs ranged from 46 to 60 feet, HWs were 32 and 39 feet).

Stomach Contents and Blubber Thickness

Stomach contents were noted for 71 individuals representing Fin, Humpback, and Sperm whales, all taken during the Coal Harbour era. All Fin whales (n=49) and Humpback whales (n=20) sampled contained euphausiids (undetermined species), while the two Sperm whales whose stomach contents were examined contained Robust Clubhook Squid (*Moroteuthis robusta),* unidentified squid, and miscellaneous fish species.

Blubber thickness was documented for 68 individuals (59 FW, 5 HW, 2 SPW, 1 SW). The location on the body where the blubber was measured is undocumented, and should be interpreted accordingly. Average, minimum and maximum blubber thickness for each species is summarized in Table 10.

Discussion

In addition to the considerations below, see the main text for discussion of the implications of these records for the historical importance of this area for fin whales as well as fin whale – humpback whale interactions.

The analysis of historical whaling records, early explorers’ logs, and review of local First Nation cultural indicators of whales indicate that the Caamaño Sound area has had notable concentrations of large whales for at least the past two or three centuries, but these aggregations were likely

present long before early explorers arrival, as indicated by the knowledge, use, prevalence and importance of whales in the cultures of the Gitga'at and Gitxaala people. Some of the cultural practices involving whales could only have developed over long time periods living with whales (inclusion of whale materials in ceremonial clothing, hunting whales (Gitxaala), specific words in vocabulary, traditional place names with whale focus, etc). These people have lived in this region for millennia, it is plausible the whales in the region have also inhabited this area for as long or longer (deglaciation of the study area occurred approx. 15,500 – 13,000 years before present; Shaw et al. 2017). Whalers knew about and exploited the area's whale abundance intentionally and routinely over a number of decades in the more recent whaling periods. A substantial number of Fin whales were taken in Caamaño Sound and associated inner coastal waterways--many more than any other adjacent nearshore areas of BC over the entire whaling period. This historical information supports the current idea that Caamaño Sound is unique, at least on the BC coast, in having consistent feeding aggregations of Fin whales in nearshore coastal waterways. The take of a North Pacific Right whale only 50 miles from Caamano Sound during the early whaling period also suggest that one of the most endangered populations of large whale in existence today was at least occasionally present in the Caamaño Sound area just less than two centuries ago.

Also of great interest are the catches of four Sperm whales in Caamaño Sound. Sperm whales are only occasionally observed in inshore coastal waters in some parts of the world, including Southeast Alaska (Straley and Wild 2015), where a few repeat individuals began using Chatham Strait relatively recently and have continued to do so, potentially motivated by depredation opportunities (SEASWAP 2017). In British Columbia, the deep waters of Moresby Trough lead from preferred Sperm whale habitat on the shelf break south of Haida Gwaii (Ford et al 2014) to the mouth of Caamaño Sound, creating a path of deep water for Sperm whales to venture towards Caamaño. Inside Caamaño and waters around Gil Island, the whales would find the rich and very deep waters of Squally and Whale Channels. Although these features may partially explain these whales' historical presence in Caamaño Sound, it is uncertain whether Sperm whales were once regular visitors to the inner waters of Caamaño Sound. No Sperm whales have been documented in or near Caamaño Sound since. It is possible that just a few individuals learned to use the area, and with their deaths, this tradition was lost.

There are still several questions about the actual logistics of whaling in Caamaño Sound that remain to be answered. Did the Caamaño Sound area have a reputation for consistently producing catches of preferred species in sheltered waters? Was the area used primarily when weather was too poor offshore in the main whaling grounds? At what point during the whaling period in BC did whalers know that Caamaño Sound was a worthwhile hunting ground, and what observations led to that idea? Were certain boats allocated to hunt there each season, or was it a preference of certain whaling captains? *West Whale 4* killed numerous whales in Caamaño Sound in 1963, but only went to Caamaño Sound once in all of the 1964 season. Did the captain from 1963 change vessels, or was there rumour of a lack of whales in Caamaño in 1964, or another reason for this change? Did the spotting plane used by the Coal Harbour whaling station ever fly to Caamaño to confirm when whales were present prior to a vessel travelling the nearly 200 miles without guarantee of a catch? Did the whalers have any interactions or agreements with the First Nation people in the area?

There are also many questions about the Gitga'at and Gitxaala cultures as they relate to whales that would be informative. Are there any words specific to the species of large whales in their territories? The Gitga'at are one of few northwest coast Nations with a Killerwhale Clan – does this relate to killer whale presence in their territory?

Caamaño Sound is located in a remote wilderness region that has only received research attention focused on its cetacean species in the past three decades. Even with a short study history,

research has documented the extensive use of this area by several SARA-listed whale species, and has highlighted its importance to these populations (Nichol and Ford 2012, Ford 2006, Ford et al. 2013, DFO 2009, Ashe et al. 2013, Keen et al. 2017, etc.). Currently, this area is receiving considerable attention from industry with several proposed development projects that would require large-scale increases in shipping traffic. Ship-strikes with large whales, a substantial increase in underwater noise, the potential for environmental disaster from oil spills, and the inherent disturbance such traffic would introduce to this wilderness area are significant concerns when considering such proposals and their impacts on threatened cetacean populations in this area.

Knowledge of the historical whale presence here provides the perspective that this habitat has supported populations of large whales prior to written history for the area as indicated by the depth to which whales are embedded in the culture of local First Nation groups, as well as oral accounts shared with early explorers. This perspective, in combination with our current knowledge on large whales that use the area, suggests that whale populations using Caamaño Sound are still recovering from whaling efforts. Four species caught by whalers in this area (Sperm, Blue, Sei, and Right) have not been seen in this area since whaling, but there is potential they will return in the future as these populations recover.

Acknowledgments (JP)

I would like to thank Eva Stredulinsky for helping retrieve the data from the historical whaling database and helping with the analysis, as well as her efforts in proofreading several editions of the document. Brian Gisborne provided much appreciated insight into the early explorations of the area, which helped focus my efforts, and also provided the published log of Captain James Colnett. Thank you to the Cetacean Research Program, specifically Linda Nichol, at the Pacific Biological Station for helping create and maintain the B.C Historical Whaling Database, from which much of the data in this report came. I would also like to express my gratitude to the late Ernie Hill for sharing information on the historical use of whale carcasses by the Gitga'at First Nation. Archie Dundas and Nicole Clifton helped find and retrieve the whaling harpoon in Hartley Bay that was photographed in this report. I would also like to thank the staff at the Royal BC Archives for their assistance in helping me find and access copies of several explorers' journals archived there.

Table 1. Numbers of individual whales taken in Caamaño Sound (n=129) by species and year by the two BC shore stations responsible for the whaling that took place in Caamaño Sound, Rose Harbour and Coal Harbour. Species codes are as follows: FW= Fin, HW=Humpback, SW = Sei, BW = Blue, SPW = Sperm.

	Rose Harbour				Coal Harbour				
**YEAR**	FW	HW	SW*	BW	FW	HW	SW	BW	SPW
1926	0	0	0	1					
1927	6	2	1	0					
1954					3	3	0	0	0
1955					12	3	0	0	1
1956					8	3	0	0	0
1957					3	3	0	0	0
1958					1	3	0	0	0
1959					2	1	0	0	0
1963					33	2	0	0	0
1964					5	2	0	0	0
1965					13	3	1	0	0
1966					11	0	0	0	2
1967					0	0	0	0	1
TOTAL	6	2	1	1	91	23	1	0	4

* Misidentification between Fin and Sei whales during the Rose Harbour era was likely a regular occurrence, and Sei whale numbers from Rose Harbour area may be questionable.

Table 2: Number of whales sighted by whaling vessels per year, for each species. Note: sighted animals are often also killed after being sighted, as was the case with the SW and SPW.

**Year**	**FW**	**HW**	**SW**	**BW**	**SPW**	**Total**
1963	132	7	0	0	0	139
1964	28	2	0	0	0	30
1965	45	2	2	0	0	49
1966	51	0	0	0	4	55
TOTAL	256	11	2	0	4	**273**

Table 3: Number of individuals taken by species per month, including monthly totals.

**Month**	**FW**	**HW**	**SW**	**SPW**	**BW**	**TOTAL**
April	0	0	0	0	0	0
May	0	1	2	0	0	3
June	7	0	0	1	0	8
July	17	3	0	1	0	21
August	51	10	0	2	1	64
September	22	11	0	0	0	33

Table 4: Number of individuals sighted by species per month, including monthly totals. Note: whalers may have been biased towards recording sightings of desirable species.

Month	FW	HW	SW	BW	SPW	Total
April	0	0	0	0	0	0
May	0	0	2	0	0	2
June	12	0	0	0	0	12
July	44	0	0	0	0	44
August	123	8	0	0	4	135
September	77	3	0	0	0	80

Table 5: Summary of monthly effort presented in boat days, kills per month, and monthly CPUE.

Month	Boat Days	Kills	CPUE
May	3	3	1.0
June	7	8	1.1
July	12	21	1.8
August	45	64	1.4
September	20	33	1.7

Table 6: Summary of annual effort presented in boat days, kills per year, and annual CPUE.

Year	Boats	Boat Days	Kills	CPUE
1926	1	1	1	1.0
1927	4	8	9	1.1
1954	2	4	6	1.5
1955	3	8	16	2.0
1956	3	6	11	1.8
1957	3	5	6	1.2
1958	2	2	4	2.0
1959	3	3	3	1.0
1963	4	24	35	1.5
1964	2	4	7	1.8
1965	4	13	17	1.3
1966	4	8	13	1.6
1967	1	1	1	1.0
Average	2.8	6.7	9.9	1.4

Table 7: Names of the thirteen ships that are known to have operated in Caamaño Sound as evidenced by at least one kill made there. The table also indicates kills by species for each ship.

Catcher Ship Name	BW	FW	HW	SW	SPW	TOTAL
S.S. Brown	1	5	0	0	0	6
S.S. Blue	0	0	1	0	0	1
S.S. Black	0	0	1	1	0	2
S.S. White	0	1	0	0	0	1
Lavallee	0	15	2	0	0	17
Nahmint	0	0	5	0	0	5
West Whale 1 (Polar V)	0	0	2	0	0	2
West Whale 2 (Globe VII)	0	3	3	0	1	7
West Whale 4 (Tahsis Chief)	0	35	4	0	2	41
West Whale 5	0	0	2	0	0	2
West Whale 6	0	4	0	0	0	4
West Whale 7	0	18	2	1	0	21
West Whale 8	0	16	3	0	1	20

Table 8: Mean, maximum, and minimum lengths by species in feet. Numbers of whales killed for each species by sex.

Species	Length (ft)			Sex	
	Mean	Max	Min	Female	Male
Fin Whale	58	68	45	57	40
Humpback	39.7	47	29	12	13
Sperm Whale	56.7	53	50	0	4
Sei Whale	41	42	40	0	2
Right Whale	55.0	55	55	1	0

Table 9: Mammary condition for female Fin and Humpback whales killed in Caamaño Sound. The number of individuals in each condition category is given. Descriptoins taken from Nichol et al. 2002: *Lactating*: contained traces of milk; *Nonparous*: immature females, those pregnant for the first time, those that have ovulated but have not yet become pregnant; *Involuted*: Pregnant for second or third, etc., time but presently resting, or those that have started to swell in preparation for lactating or those that have begun to contract (i.e. just finished lactating). *Parous*: definition not found in original data, however common usage of "parous" refers to animals that have previously given birth, so we assume this indicates females with birthing experience who were not pregnant or lactating at the time of their capture; *Immature*: description not found in original data, however we assume this indicates females that have not yet ovulated.

Species	Lactating	Nonparous	Involuted	Parous	Immature	n
Fin Whale	2	12	7	0	0	21
Humpback	0	2	0	0	0	2

Table 10: Blubber thickness measured in centimeters as documented for 68 whales caught in Caamaño Sound.

Species	Min	Max	Average	n
FW	4.1	13	7.4	59
HW	7	11.5	7.5	5
SPW	12	13	12.6	3
SW	6.9	6.9	6.9	1

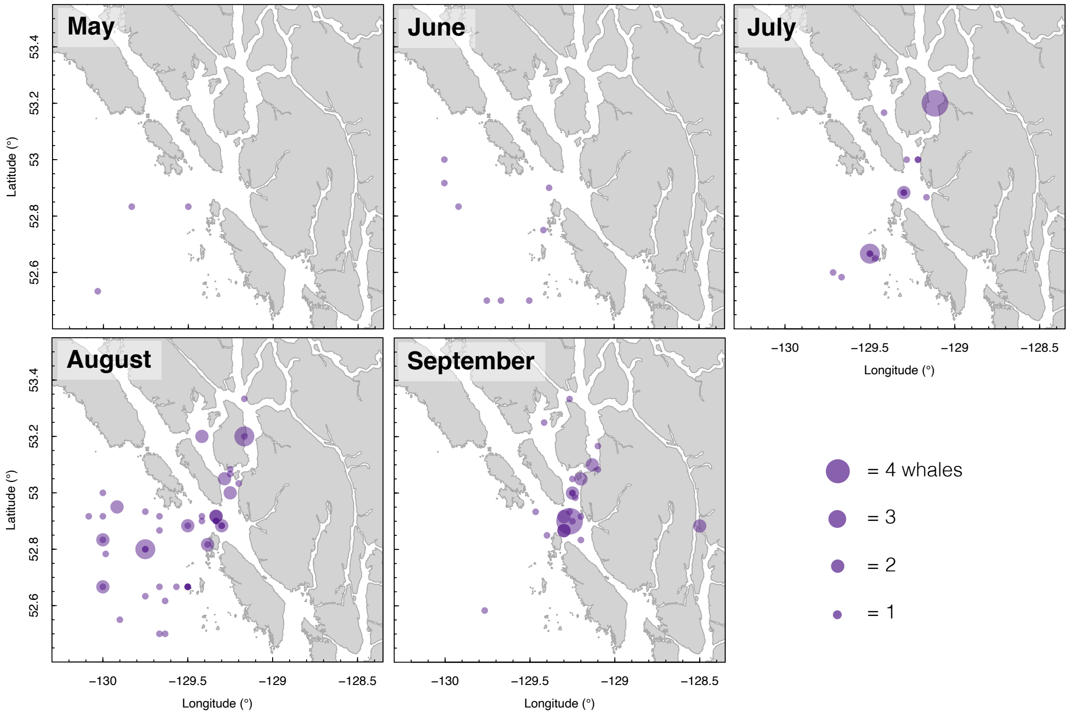

Figure 2: Catch locations by month. The size of the dot represents the number of animals that were killed in the event at that location.

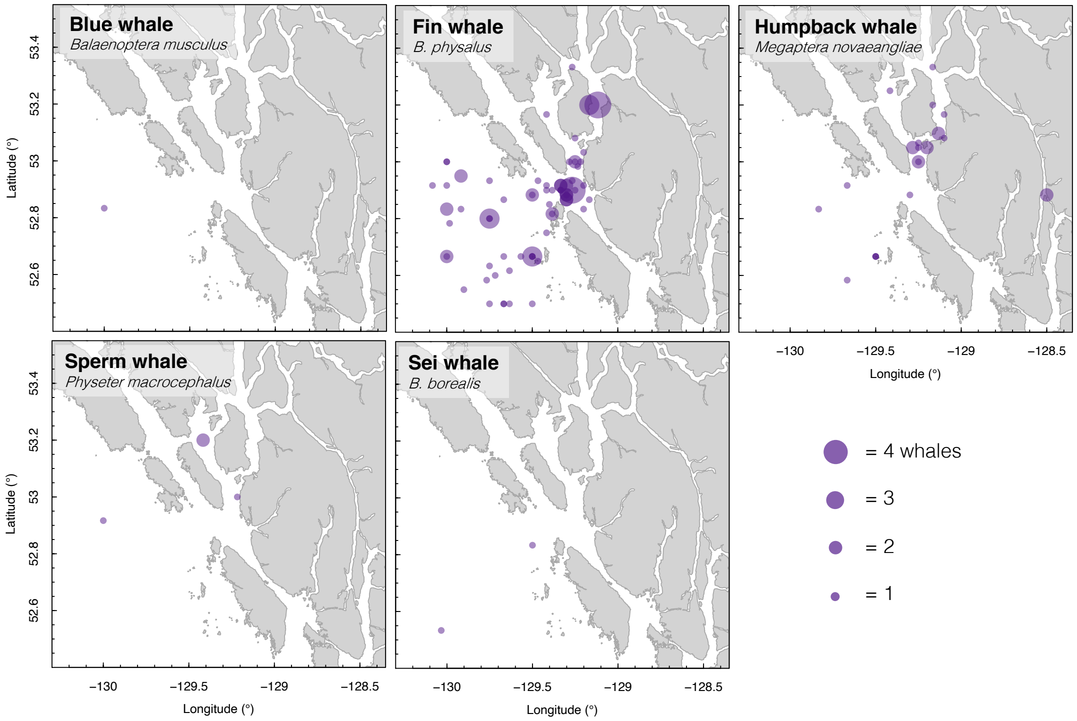

Figure 3: Catch locations of the five species caught in the study area. Dot size is proportional to the number of animals killed in the event at that location.

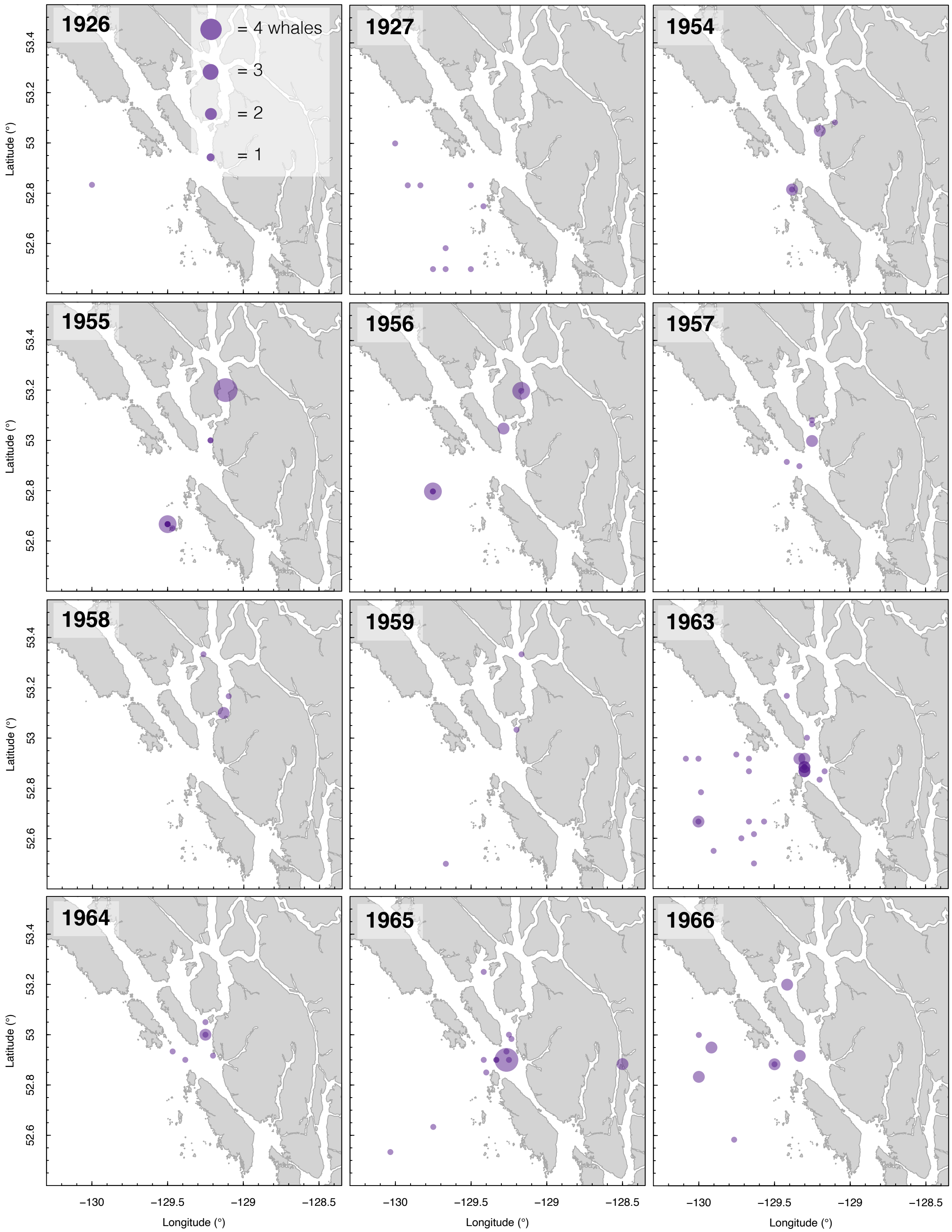

Figure 4: Catch locations for each year that whaling was conducted in the Caamaño Sound area.

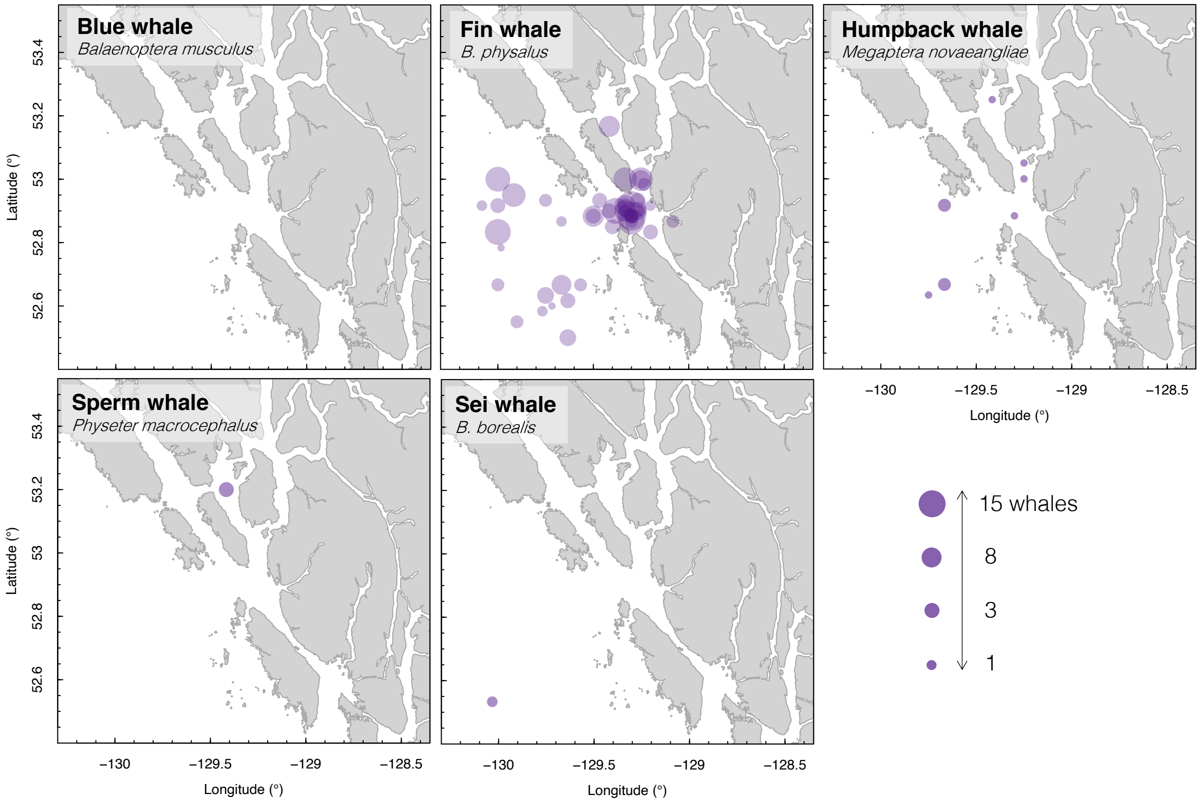

Figure 5: Sighting locations of the five species caught in the study area. Dot size is proportional to the number of animals sighted in the event at that location. Note that no Blue Whale sightings were logged.

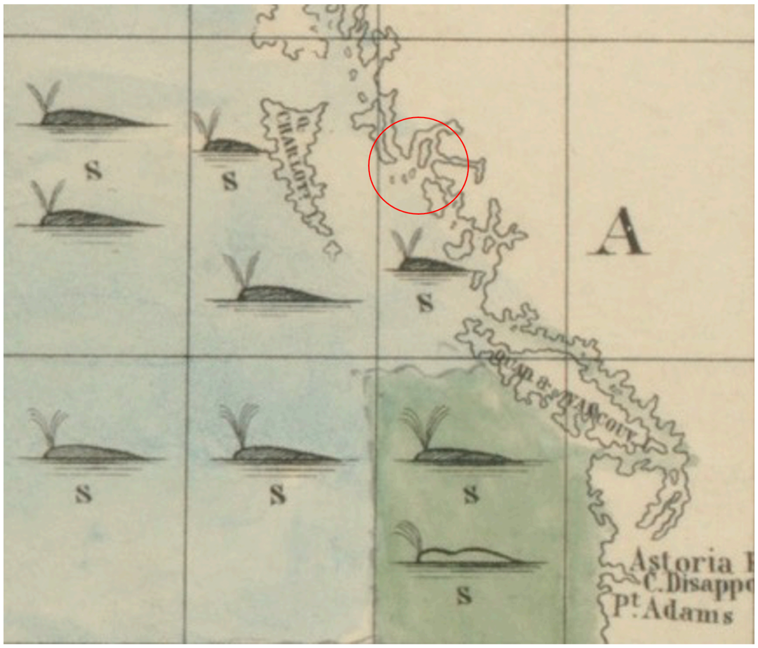

Figure 6: A cropping of M.F Maury's Whale Chart Series F (1851), showing the coast of British Columbia. Note the symbol for Right whale ('V'-shaped spout) in Queen Charlotte Sound adjacent Caamaño Sound (red circle).

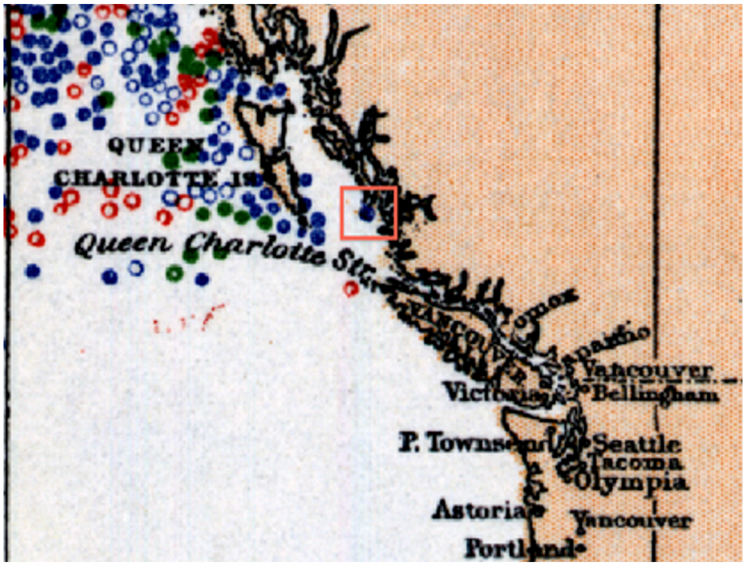

Figure 7: A cropping of Townsend's Chart, showing the coast of British Columbia and the North Pacific Right whale catch location just south of Caamaño Sound off Price Island.

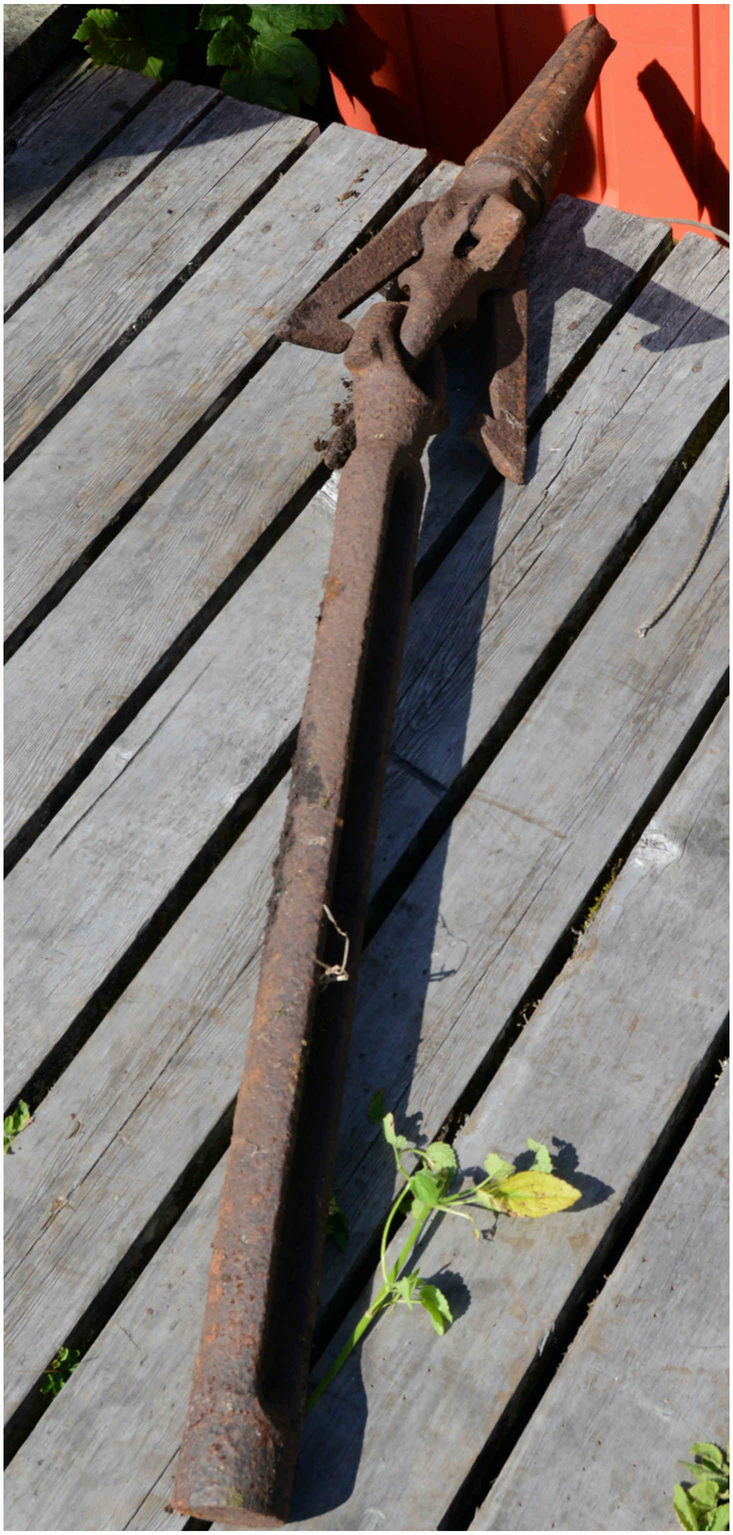

Figure 8: Several people from Hartley Bay found this whaling harpoon fifteen years ago while beach combing on the west coast of Campania Island in Caamaño Sound. The explosive tip is undetonated, allowing for a good look at the tip and articulating flukes. One has to wonder how the harpoon ended up on the beach; was it inside a lost whale carcass that drifted ashore, or possibly an errant shot that skipped off the waters' surface?

References

Ashe E, Wray J, Picard CR, Williams R. (2013) Abundance and survival of Pacific humpback whales in a proposed critical habitat area. PLoS One. 8: e75228.

Beland, S.L (2014). Learning from the past: genetic analyses of ancient and contemporary samples identify how historic and pre-historic events have shaped modern whale populations. Msc Thesis. St. Mark's University. 94 pp.

DFO. 2009. Recovery Potential Assessment for West Coast Transient Killer Whales. DFO Can. Sci. Advis. Sec. Sci. Advis. Rep. 2009/039.

Fisheries and Oceans Canada. 2013. Recovery Strategy for the North Pacific Humpback Whale (Megaptera novaeangliae) in Canada. Species at Risk Act Recovery Strategy Series. Fisheries and Oceans Canada, Ottawa. X + 67 pp.

Ford J.K.B, Durban J.W., Ellis G.M., Towers J.R., Pilkington J.F., Barrett-Lennard L.G., Andrews R.D. 2012. New Insights into the northward migration route of gray whales between Vancouver Island, British Columbia, and southeastern Alaska. Marine Mammal Science 29(2):325-337.

Ford J.K.B, Stredulinsky E, Towers J.R., Ellis G.M. 2013. Information in Support of the Identification of Critical Habitat for Transient Killer Whales (Orcinus orca) off the West Coast of Canada. Canadian Science Advisory Secretariat Research Document 2012/155. Iv + 46 p.

Ford J.K.B. 2006. An Assessment of Critical Habitats of Resident Killer Whales in Waters off the Pacific Coast of Canada. Canadian Science Advisory Secretariat Research Document 2006/072: vi + 34 p.

Galois, R. 2005, A Voyage of the North West Side of America: The Journals of James Colnett, 1786-89. University of British Columbia Press.

Gregr E.J., Nichol L., Ford J.K.B, Ellis G., Trites A.W. 2000. Migration and population structure of Northeastern Pacific whales off British Columbia: An analysis of commercial whaling records from 1908-1967. Marine Mammal Science, 16(4):699-727.

Keen EM, Wray J, Meuter H, Thompson K-L, Barlow JP, Picard CR. (2017) "Whale wave": shifting strategies structure the complex use of critical fjord habitat by humpbacks. Marine Ecology Progress Series 567: 211–233.

Marsden, S. 2012. The Gitk'a'ata, their History, and there Territories: Report Submitted to the Gitk'a'ata.

Maury, M.F. (1851) Investigations of the Winds and Currents of the Sea. National Observatory. C. Alexander Press.

Maury, M.F. (1887) Citation not found.

Menzies C.R. 2011. Gitxaala Use and Occupancy in the area of the proposed Northern Gateway Pipeline Tanker Routes: Prepared on behalf of the Gitxaala Nation.

Menzies, A. (1793). Journals of Archibald Menzies. BC Archives. Accessed in 2014.

Monks, GG, AD McMillan, DE St. Claire. (2001) Nuu-Chah-Nulth Whaling: archaeological insights into antiquity, species preferences, and cultural importance. Arctic Anthropology 38(1): 60-81.

Nichol, L.M., Gregr E.J., Flinn R., Ford J.K.B, Gurney R., Michaluk L., and Peacock A. 2002. British Columbia Commercial Whaling Catch Data 1908-1967: A Detailed Description of the B.C. Historical Whaling Database. Canadian Technical Report of Fisheries and Aquatic Sciences. 2396: viii + 76 p.

Nichol, L.M., and J.K.B. Ford. 2012. Information relevant to the assessment of critical habitat for Blue, Fin, Sei and North Pacific Right Whales in British Columbia. DFO Can. Sci. Advis. Sec. Res. Doc. 2011/137. vi + 31 p.

Pender, D. (1866-68) Journals of Captain Daniel Pender. BC Archives. Accessed in 2014.

Roe, M. 2016. The Journal and Letters of Captain Charles Bishop. Routledge, 2 Park Square, Milton Park, Abingdon, Oxon 0X14 4RN

Satterfield T., Robertson L., Turner N., Pitts A. 2012. Being Gitka'a'ata: A Baseline Report on Gitka'a'ata Way of Life, a Statement of Cutural Impacts Posed by the Northern Gateway Pipeline, and a Critique of the ENGP Assessment Regarding Cultural Impacts.

Shaw J., Stacey C.D., Wu Y., Lintern D.G. 2017. Anatomy of the Kitimat fiord system, British Columbia. Geomorphology. 293 (2017) 108-129.

Townsend C.H. 1935 The Distribution of Certain Whales as Shown by Logbook Records of American Whaleships. Zoologica (NY):: 1-50+6 Charts.

Wagner H.R., Newcombe W.A. 1938. The Journal of Jacinto Caamano Part I (translated by Captain Harold Grenfell R.N). The British Columbia Historical Quarterly July 1938, 189-222.

Wagner H.R., Newcombe W.A. 1938. The Journal of Jacinto Caamano Part II (translated by Captain Harold Grenfell R.N). The British Columbia Historical Quarterly: October 1938, 264-303.

Webb R.L. 1988. On the Northwest: commercial whaling in the Pacific Northwest, 1790-1967. University of British Columbia Press Pacific.
