# Supplementary material for: Fin whales of the Great Bear Rainforest: Balaenoptera physalus velifera in a Canadian Pacific fjord system
Source: PLoS One. 2021 Sep 3;16(9):e0256815. doi: 10.1371/journal.pone.0256815 (PMC8415578; doi:10.1371/journal.pone.0256815)
Supplement: S1 File — (DOCX) [file pone.0256815.s002.docx]

**Supplementary Tables and Figures**

**Fin whales of the Great Bear Rainforest: *Balaenoptera physalus velifera* in a Canadian Pacific fjord system**

Eric M. Keen, James Pilkington, Éadin O’Mahony, Kim-Ly Thompson, Ben Hendricks, Nicole Robinson, Archibald Dundas, Linda Nichol, Hussein Alidina, Hermann Meuter, Chris R. Picard, Janie Wray

**Table S1**. Recapture statistics for identified fin whales in our study. *Left*: The number of whales seen in at least 1, 2, … 10 years. *Right*: The number of whales seen in at least 1, 2, . . . 37 encounters.

| **Recaptures** | | | | |
| --- | --- | --- | --- | --- |
| Years | Individuals |  | Encounters | Individuals |
| 1 | 83 |  | 1 | 83 |
| 2 | 37 |  | 2 | 56 |
| 3 | 27 |  | 3 | 43 |
| 4 | 18 |  | 4 | 35 |
| 5 | 12 |  | 5 | 31 |
| 6 | 5 |  | 10 | 18 |
| 7 | 4 |  | 15 | 6 |
| 8 | 1 |  | 21 | 4 |
| 9 | 1 |  | 28 | 2 |
| 10 | 0 |  | 37 | 1 |

**Table S2.** Candidate POPAN models for estimating fin whale abundance based on photo-id mark-recapture. Models are ranked by AICc performance, and the highest-performing models are in boldface. Model parameters included apparent survival ($\phi$), recapture probability (*p*), and entry probability (*p_ent_*), and were modeled variously as constant (*) or varying over time (*t*).

| $\phi$ | *p* | *p_ent_* | Parameters | AICc |
| --- | --- | --- | --- | --- |
| ******* | ******* | ***t*** | **11** | **475.11** |
| ******* | ***t*** | ***t*** | **21** | **476.11** |
| *t* | *** | *t* | 19 | 481.52 |
| *t* | *t* | *t* | 27 | 493.6 |
| *** | *** | *** | 4 | 505.49 |

**Table S3.** Recapture rates for each dorsal distinctiveness category. 1 = Extremely distinctive, 2 = adequately distinctive; 3 = indistinct and difficult to identify.

| **Year** | **Return rate (%) by dorsal distinctiveness** | | | | |
| --- | --- | --- | --- | --- | --- |
|  | 1 | 2 | 3 | 1-3 | 2-3 |
| 2006 | 0 | 0 | 0 | 0 | 0 |
| 2007 | 0 | 0 | 0 | 0 | 0 |
| 2008 | 0 | 0 | 0 | 0 | 0 |
| 2009 | 17 | 33 | 0 | 15 | 14 |
| 2010 | 50 | 0 | 0 | 40 | 0 |
| 2011 | 25 | 0 | 29 | 24 | 25 |
| 2012 | 100 | 50 | 67 | 80 | 62 |
| 2013 | 39 | 20 | 50 | 39 | 42 |
| 2014 | 90 | 60 | 75 | 76 | 77 |
| 2015 | 90 | 50 | 75 | 77 | 71 |
| 2016 | 82 | 50 | 75 | 76 | 75 |
| 2017 | - | - | - | - | - |
| 2018 | - | - | - | - | - |
| 2019 | 71 | 100 | 100 | 85 | 100 |
| **Mean**  2012 – 2019 | 79 | 55 | 74 | 72 | 71 |
| **SD** | 22 | 25 | 16 | 17 | 19 |

**Table S4.** Habitat use statistics for the population of fin whales identified in our study. For each metric, statistics are given for all identified whales (subset > 0), as well as for all whales seen in more than one year (subset > 1).

| **Metrics** | **Subset** | **Mean** | **SD** | **Median** | **Min.** | **Max.** |
| --- | --- | --- | --- | --- | --- | --- |
| Years seen | > 0 | 2.3 | 1.8 | 1 | 1 | 9 |
|  | > 1 | 3.8 | 1.8 | 3 | 2 | 9 |
| Encounters | > 0 | 5.6 | 6.5 | 3 | 1 | 37 |
|  | > 1 | 10.2 | 7.3 | 9 | 2 | 37 |
| Earliest first observation (doy) | > 0 | 198 | 34 | 203 | 77 | 300 |
|  | > 1 | 183 | 36 | 180 | 77 | 254 |
| Latest first observation (doy) | > 0 | 238 | 33 | 241 | 153 | 300 |
|  | > 1 | 260 | 26 | 263 | 153 | 300 |
| Average first observation (doy) | > 0 | 210 | 28 | 213 | 115 | 300 |
|  | > 1 | 210 | 29 | 213 | 155 | 277 |
| Average last observation (doy) | > 0 | 228 | 29 | 231 | 125 | 300 |
|  | > 1 | 235 | 27 | 238 | 125 | 277 |
| Average stay (days) | > 0 | 18 | 23 | 5 | 1 | 110 |
|  | > 1 | 25 | 22 | 23 | 1 | 110 |
| Occupancy (IO) | > 0 | 0.056 | 0.098 | 0.028 | 0.000 | 0.667 |
|  | > 1 | 0.071 | 0.078 | 0.047 | 0.000 | 0.393 |
| Permanence (IT) | > 0 | 0.153 | 0.211 | 0.053 | 0.000 | 1.000 |
|  | > 1 | 0.208 | 0.174 | 0.210 | 0.000 | 0.708 |
| Periodicity (It) | > 0 | 0.070 | 0.149 | 0.025 | 0.000 | 1.000 |
|  | > 1 | 0.071 | 0.113 | 0.042 | 0.000 | 0.667 |
| Standardized Site Fidelity Index | > 0 | 0.104 | 0.174 | 0.048 | 0.000 | 1.000 |
|  | > 1 | 0.110 | 0.135 | 0.078 | 0.000 | 0.778 |
| Interannual return rate  (total study) | > 0 | 0.189 | 0.153 | 0.083 | 0.083 | 0.750 |
|  | > 1 | 0.320 | 0.146 | 0.250 | 0.167 | 0.750 |
| Interannual return rate  (since year first seen) | >0 | 0.410 | 0.270 | 0.333 | 0.083 | 1.000 |
|  | > 1 | 0.593 | 0.229 | 0.600 | 0.167 | 1.000 |

**Table S5.** Model fits of candidate fin whale detection functions based upon line transect analysis of Bangarang surveys, 2013 – 2015.

| **Key function** | **cosin adjustment** | **C-vM**  **p-value** | $\bar{\boldsymbol{P}_{\boldsymbol{a}}}$ | **S.E.** $\bar{\boldsymbol{P}_{\boldsymbol{a}}}$ | **∆AICc** |
| --- | --- | --- | --- | --- | --- |
| **Half-normal** | **No** | **0.8554** | **0.5067** | **0.0684** | **0.0000** |
| Half-normal | Yes | 0.8554 | 0.5067 | 0.0684 | 0.0000 |
| Hazard rate | No | 0.9631 | 0.4699 | 0.0885 | 2.0269 |
| Hazard rate | Yes | 0.2063 | 0.6829 | 0.0611 | 3.0554 |

**Table S6.** Results of line transect analyses of systematic surveys for fin whales in 2013 – 2015.

|  | **Stratum** | | | | |  |
| --- | --- | --- | --- | --- | --- | --- |
|  | *1*  *Caamano Sound* | *2*  *Estevan Sound* | *3*  *Campania Sound* | *4*  *Squally Channel* | *5*  *Inland waters* | **Total** |
| Transects | 11 | 10 | 20 | 31 | 47 | 119 |
| Effort (km) | 224 | 189 | 343 | 584 | 985 | 2,324 |
| Area (km^2^) | 289 | 153 | 112 | 221 | 439 | 1,214 |
| Covered area (km^2^) | 952 | 804 | 1,461 | 2,489 | 4,195 | 9,901 |
|  |  |  |  |  |  |  |
| Detections | 7 | 1 | 7 | 23 | 0 | 38 |
| Encounter rate | 0.0313 | 0.0053 | 0.0204 | 0.0394 | 0.0000 | 0.0163 |
| SE | 0.0121 | 0.0059 | 0.0113 | 0.0094 | 0.0000 | 0.0034 |
| CV | 0.3859 | 1.1080 | 0.5562 | 0.2387 | 0.0000 | 0.2106 |
|  |  |  |  |  |  |  |
| Cluster size | 1.8571 | - | 1.8571 | 2.0434 | - | 1.9192 |
| SE | 0.3357 | - | 0.4281 | 0.1700 | - | 0.1748 |
| CV | 0.1808 | - | 0.2305 | 0.0830 | - | 0.0852 |
|  |  |  |  |  |  |  |
| Cluster density | 0.0145 | 0.0025 | 0.0095 | 0.0182 | 0.0000 | 0.0076 |
| SE | 0.0059 | 0.0027 | 0.0054 | 0.0050 | 0.0000 | 0.0018 |
| CV | 0.4089 | 1.1162 | 0.5724 | 0.2742 | 0.0000 | 0.2356 |
| LCL | 0.0062 | 0.0003 | 0.0031 | 0.0106 | 0.0000 | 0.0048 |
| UCL | 0.0340 | 0.0186 | 0.0286 | 0.0313 | 0.0000 | 0.0120 |
|  |  |  |  |  |  |  |
| Clusters at once | 4.1905 | 0.3825 | 1.0640 | 4.0222 | 0.0000 | 9.2264 |
| LCL | 1.7918 | 0.0459 | 0.3472 | 2.3426 | 0.0000 | 5.8272 |
| UCL | 9.8260 | 2.8458 | 3.2032 | 6.9173 | 0.0000 | 14.5680 |
| Individuals at once | 7.7822 | 0.7103 | 1.9760 | 8.2189 | 0.0000 | 17.7073 |
| LCL | 3.3276 | 0.0852 | 0.6448 | 4.7869 | 0.0000 | 11.1836 |
| UCL | 18.2479 | 5.2742 | 5.9487 | 14.1348 | 0.0000 | 27.9589 |

**Notes:**

Stratum areas are drawn from Keen (2017).

Detections include only those clusters within the truncation distance (i.e., excluding the furthest 10% of detections).

Since Estevan had only one sighting, its mean cluster size was assumed to be the same as Caamano and Estevan.

‘Individuals at once’ is estimated using the mean cluster size in each stratum.

**Table S7.** Fin whale group sizes observed in the Kitimat Fjord System.

|  |  | Group size | | |
| --- | --- | --- | --- | --- |
| Research mode | Groups | *Median* | *Mean* | *SD* |
| All | 1,906 | 2 | 1.97 | 1.31 |
| Vessel-based | 601 | 2 | 2.13 | 1.53 |
| Land-based | 1,305 | 2 | 1.89 | 1.18 |

**Table S8.** Dyad recapture statistics for pairs of associated fin whales in our study. *Left*: The number of dyads seen in at least 1, 2, … 4 years. *Right*: The number of dyads seen in at least 1, 2, . . . 10 encounters.

| **Dyad recaptures** | | | | | | |
| --- | --- | --- | --- | --- | --- | --- |
| *Years* | *Dyads* | *Individuals* |  | *Encounters* | *Dyads* | *Individuals* |
| 1 | 331 | 78 |  | 1 | 331 | 78 |
| 2 | 36 | 24 |  | 2 | 69 | 41 |
| 3 | 9 | 10 |  | 3 | 18 | 20 |
| 4* | 2 | 3 |  | 4 | 7 | 10 |
|  |  |  |  | 5 | 1 | 2 |
|  |  |  |  | 10** | 1 | 2 |

* fw0001 – fw0026, fw0001 – fw0008

** fw0001 – fw0008


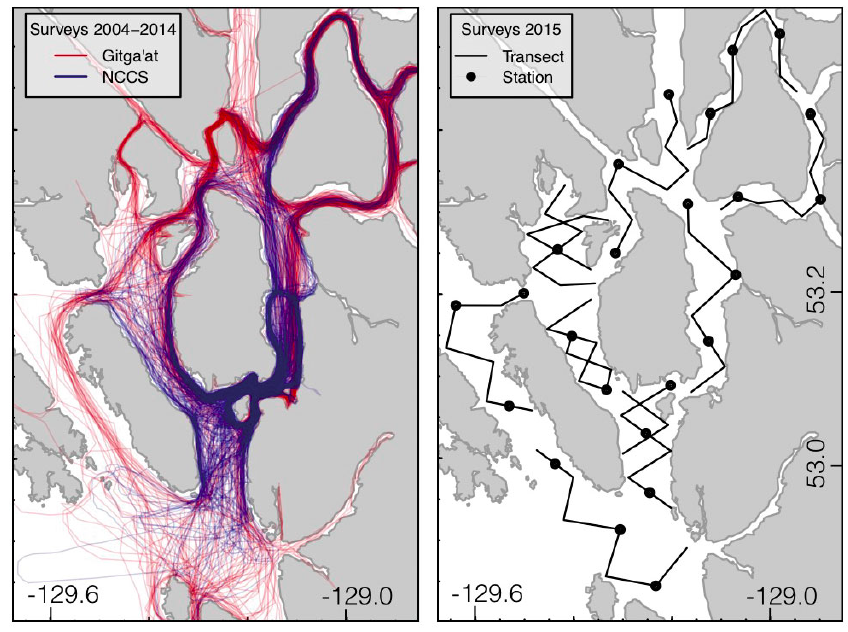


**Figure S1.** Left: Survey effort by Gitga’at (red) and North Coast Cetacean Society (NCCS, *Elemiah*, blue), 2005 to 2014. Right: Sampling plan for 2015 vessel transects (lines) and oceanographic stations (filled circles) in 2015 aboard *RV Bangarang*. Reprinted from Keen et al. (2017) ©Inter-Research 2017. Reproduced with permission to be issued here under the Creative Commons by Attribution License (CC BY 4.0).


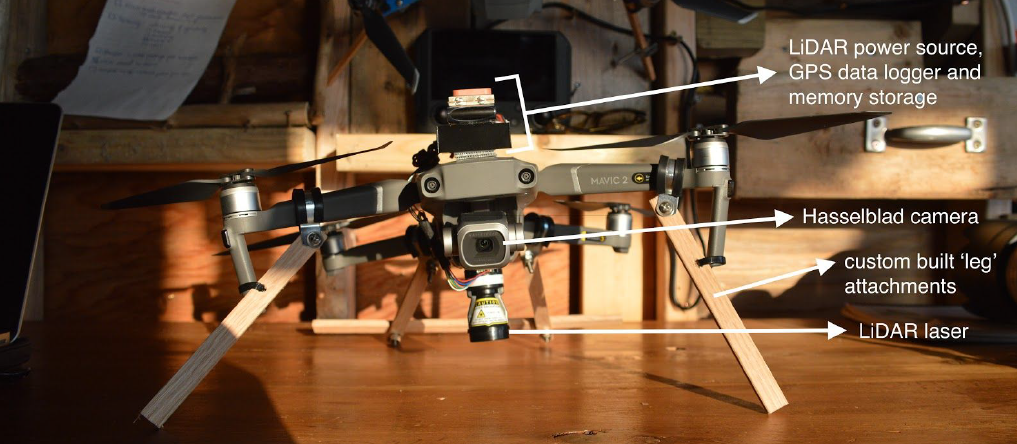


**Figure S2.** DJI Mavic 2 Pro UAS with attached LiDAR/GPS data logger (Lightware SF11 lidar, https://lightwarelidar.com) and custom built ‘leg’ attachments to create space for the laser and provide a handle to catch the UAS in flight.


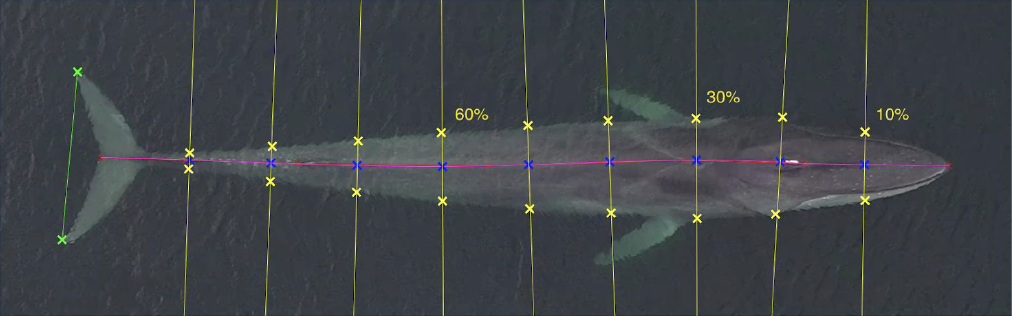


**Figure S3.** Markings of measurements taken from each video still, using Graphical User Interface ‘Whalength v5’ developed by (Dawson et al. 2017) . When the whale’s sides were obscured by disturbed water, measurements at that increment were not taken.

**Figure S4.** Reported locations of fin whale catches in (solid dots) and near (open dots) the Kitimat Fjord System from commercial whaling, 1927-1966. Points are scaled by the number of captures at the exact same coordinate position and color-coded by sex (red = male; blue = female). Dashed orange lines indicates approximate boundary for the Kitimat Fjord System.

**Figure S5.** Maps of BC Cetacean Sightings Network detections of fin whales, by year, within (closed circles) and near (open circles) the entrance to the Kitimat Fjord System. Reports are parsed by “Probable” species identification (blue) and “Certain” (black).

**Figure S6.** Map of BC Cetacean Sightings Network detections of fin whales within (solid circles) and near (open circles) the entrance to the Kitimat Fjord System. Reports are parsed by “Probable” species identification (blue) and “Certain” (black). Dashed orange lines indicates approximate boundary for the Kitimat Fjord System.

**Figure S7**. Discovery curve, 2006 – 2019.

**Figure S8.** Lagged Identification Rate (LIR) of fin whales in the Kitimat Fjord System. Grey dots represent the LIR calculated for each time lag ($\tau$, in days) tested. Black line is the running mean of LIR (window=10 days, first point forced to the $\tau$ = 1). Blue line and shaded area represent the median and 95% confidence interval (2.5% and 97.5% quantiles), respectively, of the permutation tests (n=100). Lags at which the running mean rises above the shaded area indicate significant patterns in residency behavior.

**Figure S9.** Best-fitting detection function superimposed upon histogram of detection distances of fin whales observed during line transect survey effort aboard the *RV Bangarang* (2013 – 2015). *See Table S5 for model details.*

**Figure S10.** Seasonal detection rates of fin whales from shore-based surveys from three study areas in the Kitimat Fjord System: North Squally (top), the most inland land-based research platform at Fin Island; Central channels (middle), surveyed from Whale Point by Cetacea Lab volunteers; and South Caamano Sound (bottom), surveyed from Ulric Point (2010 and 2011) and the Wall Islets (2014).

**Figure S11.** Geographic and annual patterns in the seasonal timing of peak detection rates of fin whales from shore-based platforms. *Left:* Date of peak detection rate is pooled by study area. ‘Outer’ refers to South Caamano Sound, the outermost waterway of the fjord system (Ulric Point and Wall Islet platforms in 2010-2011 and 2014, respectively). ‘Central’ refers to Whale Point surveys in the fjord’s central channels (2011 – 2016). ‘Inner’ refers to surveys of North Squally Channel from Fin Island, the inland-most shore-based platform in this study. Right: Date of peak detection displayed by year. Each dot represents the finding of a single research platform. Dashed line represents a linear regression (p=0.005, r^2^ = 0.052).


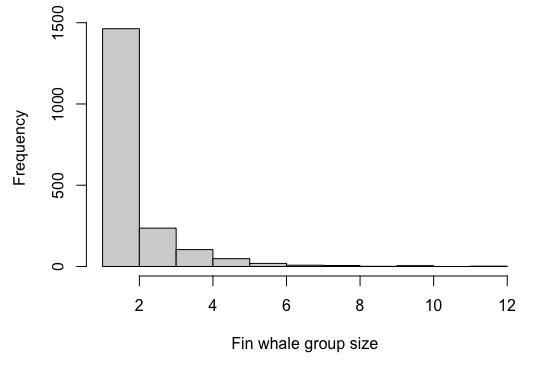


**Figure S12.** Histogram of cluster sizes observed across all platforms (n= 1,985 detections).

**Figure S13.** Geographic patterns in the effective per-capita calving rate, calculated here as the proportion of detected groups in which a calf was observed, based upon shore-based surveys. Sample sizes indicate the number of years in which calves were noted. ‘Outer’ refers to South Caamano Sound, the outermost waterway of the fjord system (Ulric Point and Wall Islet platforms in 2010-2011 and 2014, respectively). ‘Central’ refers to Whale Point surveys in the fjord’s central channels (2011 – 2016). ‘Inner’ refers to surveys of North Squally Channel from Fin Island, the inland-most shore-based platform in this study. Right: Date of peak detection displayed by year. Each dot represents the finding of a single research platform. Dashed line represents a linear regression (p=0.005, r^2^ = 0.052).
